# Net Ecosystem CO_2_ Exchange of a Subalpine Spruce Forest in Switzerland Over 26 Years: Effects of Phenology and Contributions of Abiotic Drivers at Daily Time Scales

**DOI:** 10.1111/gcb.70371

**Published:** 2025-07-24

**Authors:** Luana Krebs, Lukas Hörtnagl, Liliana Scapucci, Mana Gharun, Iris Feigenwinter, Nina Buchmann

**Affiliations:** ^1^ Department of Environmental Systems Science Institute of Agricultural Sciences, ETH Zürich Zürich Switzerland; ^2^ Department of Geosciences Institute of Landscape Ecology, University of Münster Münster Germany

**Keywords:** acclimation, carbon phenology, carbon sink, contribution of drivers, evergreen forest, NEE

## Abstract

Climate change affects carbon sequestration dynamics and phenology in forests, especially in alpine and subalpine regions. Here, long‐term trends in climate, net ecosystem CO_2_ exchange (NEE), net carbon uptake period (CUP_net_) and their drivers were investigated, using 26 years of flux measurements in a subalpine spruce forest (CH‐Dav, Switzerland; 1997 to 2022). CUP_net_ length, start (SOS) and end of season (EOS) were extracted from smoothed daily NEE time series. We used machine learning to determine the importance of environmental drivers on daily NEE and CUP_net_. Annual mean and maximum air temperatures (T_air_) increased, while soil water content (SWC) decreased significantly between 1997 and 2022. Annual C sinks increased from 1997 to 2012, leveled off between 2012 and 2015, followed by a decline. Annual NEE was strongly related to CUP_net_ length, SOS, and EOS. No significant trends in CUP_net_, SOS, or EOS were detected, most likely indicating ecophysiological acclimation, that is, physiological adjustments to changing environmental conditions over the past 26 years. We identified 48 days with significant negative trends in mean daily NEE over the 26 years, that is, stronger net C uptake or weaker net C loss, particularly in spring and autumn, but no significant positive trends. Daylength, incoming shortwave radiation (Rg), SWC, and minimum T_air_ were the main drivers of daily NEE. SOS was mainly driven by daylength and T_air_, EOS by daylength and Rg. Thus, the spruce forest benefited from higher temperature between autumn and spring, with higher net C uptake during favorable conditions and reduced C loss when winter photosynthesis compensated respiration. However, high summer temperatures increasingly limited NEE, suggesting adverse effects for subalpine *Picea abies* forests in the future. Our study demonstrated that identifying driver contributions to NEE dynamics at daily time scales allows better understanding of the complexity of climate change impacts on forest C dynamics.

## Graphical abstract

This study investigated how climate change has affected carbon uptake in a subalpine spruce forest in Switzerland over 26 years. Air temperatures rose and soil moisture declined, while the amount of carbon the forest absorbed each year increased until 2012, then stabilized and decreased. Annual carbon uptake was closely linked to the length and timing of the uptake period, though no long‐term shifts in this period were observed. Daily carbon exchange was mainly influenced by daylength, sunlight, soil moisture, and temperature. Over time, carbon uptake increased during spring and autumn, while high summer temperatures increasingly limited it.

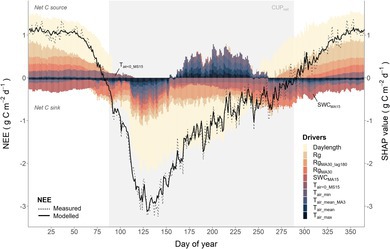

## 1 Introduction

With climate change, forests are experiencing major shifts in the environmental conditions they live in. Since climate, especially temperature and water availability, regulates plant physiological processes, including phenology, these shifts alter forest carbon (C) sequestration through changes in the net ecosystem CO_2_ exchange (NEE; Fu et al. 2017; Keenan et al. 2014). On the one hand, annual cumulative NEE may change due to alterations in daily NEE, affected by its two components, that is, gross primary production (GPP; photosynthetic CO_2_ fixation) and ecosystem respiration (R_eco_; CO_2_ respired by plants and soils), both strongly temperature‐dependent (Duffy et al. 2021; Lloyd and Taylor 1994). On the other hand, phenological changes, that is, through advances in spring phenology and/or delays in autumn phenology, might extend the growing season and thus increase the net carbon uptake period (CUP_net_) and therefore annual NEE (Chen et al. 2022; Fu et al. 2017; Keenan et al. 2014; Richardson et al. 2013). Numerous studies have reported strong relationships between CUP_net_ and annual NEE across various ecosystems (Baldocchi et al. 2005; Richardson et al. 2010, 2013; Wu et al. 2013; Wu and Chen 2013). However, the impacts of climate change on annual NEE through physiological versus phenological changes, as well as their potential climate‐carbon cycle feedbacks, remain uncertain.

High mountain areas like the Swiss Alps have experienced strong temperature increases and changes in precipitation patterns in recent decades (CH2018 2018; Hock et al. 2019), making them particularly vulnerable to climate change. Cold‐adapted subalpine forests in Switzerland are temperature‐limited and might thus benefit from a warmer climate, enhancing productivity and increasing the C sink capacity during the active season (Forkel et al. 2016; Gharun et al. 2020). However, increased ecosystem respiration rates might offset these gains by releasing more of the fixed C back into the atmosphere (Piao et al. 2008), especially during warm winters (Gharun et al. 2025). Subalpine forests might also suffer from increasing stress due to the rising prevalence and severity of droughts and pest infestations (Duveneck and Thompson 2017; Forkel et al. 2016; Gharun et al. 2020; Hammond et al. 2022). Furthermore, higher evapotranspiration due to increased temperature and vapor pressure deficit (VPD) might increase water stress and mortality in subalpine spruce forests (Lian et al. 2020; Shekhar, Hörtnagl, et al. 2024; Tresch et al. 2023). Additionally, winter warming might increase the risk of photo‐oxidative frost damage early in the growing season (Chamberlain et al. 2019). The cumulative impact of these effects on daily NEE and the annual C budget is not well understood.

Even with needles present year‐round, subalpine forests are strongly seasonality driven, with temperature and/or light limiting photosynthetic activity in winter (Gamon et al. 2016; Richardson et al. 2013). Rising temperatures may thus add warm days suitable for photosynthesis between autumn and spring, lengthening the active season and potentially enhancing the annual C sink, depending on the extent of increased respiratory losses during warmer winters (Panwar et al. 2023; Piao et al. 2007). Winter warming, however, might as well delay spring phenology by reducing chilling accumulation, because spruce trees, which have experienced a shorter period of temperatures below 4°C, have a higher heat requirement for budburst in spring (Cannell and Smith 1983; Wang et al. 2020). Unfortunately, studies on phenological shifts and their impacts on C sequestration in subalpine evergreen forests are rare. This might be due to methodological difficulties observing phenology in evergreen canopies (Korpela et al. 2023), but also to the challenge of defining a growing or active season in these trees (Körner et al. 2023). Evergreen trees can resume photosynthesis as soon as environmental conditions are favorable (Monson et al. 2005), even in winter, with cold‐adapted species reaching 30% of their full photosynthetic capacity at air temperatures as low as 0°C (Körner et al. 2023). More research on C flux phenology in cold‐adapted evergreen forests is thus needed to quantify potentially counteracting effects of phenological changes on C sequestration.

The eddy covariance (EC) method is the only technique that allows for direct and continuous measurements of NEE at the ecosystem scale, enabling us to determine whether an ecosystem acted as a net C sink (negative NEE) or a net C source (positive NEE) over a specified period (Schmid 2002). Furthermore, it offers a method to study phenology (i.e., the timing of seasonal biological events such as the C source/sink transitions in evergreen forests) apart from physiological observations (e.g., upregulation of photosynthetic capacity or changes in photoprotective mechanisms), by using CO_2_ flux phenology indicators like the net carbon uptake period (CUP_net_; Churkina et al. 2005; Panwar et al. 2023; Wu and Chen 2013). The CUP_net_ is defined as the period between the first and last zero‐crossing of NEE (Gonsamo et al. 2013). Therefore, long‐term NEE data offer the unique opportunity to evaluate climate change impacts on forest carbon uptake capacity and phenology, thereby improving our understanding of climate–carbon feedback mechanisms.

Thus, our study is based on 26 years of daily NEE data (1997–2022) from a subalpine spruce forest in Switzerland. The data set is one of the longest continuous EC time series globally, making it particularly valuable for analyzing long‐term trends in an understudied ecosystem type within FLUXNET. Here, we focused on NEE because it is the only CO_2_ flux measured directly in contrast to GPP and R_eco_, which are partitioned from NEE using statistical methods (Reichstein et al. 2005). Our objectives were to (1) identify long‐term trends in annual NEE and climate conditions; (2) determine whether changes in annual NEE were due to changes in daily NEE and/or shifts in C phenology; (3) analyze drivers of mean daily NEE as well as the start and end of CUP_net_; and (4) investigate changes in NEE drivers.

## 2 Methods

### 2.1 Study Site

The study was conducted at the ICOS Class 1 Ecosystem station Davos (CH‐Dav), located in a subalpine Norway spruce forest in the eastern Swiss Alps at an elevation of 1639 m a.s.l. (Davos Seehornwald; 46°48′55.2″N, 9°51′21.3″ E). The mean annual air temperature (T_air_) at the site was 4.3°C ± 0.7°C (1997–2022; mean ± standard deviation [SD]), the mean annual precipitation sum was 876 mm (1997–2022). See the climate chart in Figure S1 (in Supporting Information) for more details. The vegetation is dominated by Norway spruce ( *Picea abies* (L.) H.Karst), the understory is composed of blueberry dwarf shrubs ( *Vaccinium myrtillus* L. and *Vaccinium gaultherioides* Bigelow) and mosses (*Sphagnum* sp. Ehrh. and *Hylocomium splendens* (Hedw.) Schimp.), covering approx. 30% of the forest floor (Etzold et al. 2011). The average tree age in the footprint of the station is 119 years, and the average tree height is 17.5 m (Zweifel et al. 2024). Leaf area index (LAI) is around 4 (Etzold et al. 2011; ICOS ETC, n.d.). For a more detailed description of the forest structure at the study site, see Zweifel et al. (2024). Soil types are chromic cambisols and rustic podsols, and soil texture ranges from sand to sandy loam (for further details, see Jörg 2008; Krebs et al. 2024). Forest management follows the Swiss Forest Law (enacted in 1876) with a so‐called “Plenterwald” (group selection) system, cutting only small numbers of selected trees or small areas at a time (e.g., in 2006, 2016, 2020). No large‐scale forest management took place during the last decades (Burri 2023; Churakova et al. 2014).

### 2.2 Meteorological Data

We measured short‐wave radiation (Rg), air temperature (T_air_), precipitation (Prec), and relative humidity (RH) at 35 m height on top of the flux tower during the entire 26‐year study period (details on sensor types are listed in Table S1). Furthermore, photosynthetic photon flux density (PPFD) was measured on top of the flux tower starting in 2001, and soil water content (SWC) at 15 cm soil depth starting in 2007. Vapor pressure deficit (VPD) was calculated using T_air_ and RH measured at the tower. Daylength calculation for each day was done based on solar declination and latitude.

### 2.3 Eddy Covariance Data and Flux Calculations

The eddy covariance (EC) method was used to determine net ecosystem CO_2_ exchange (NEE) between the atmosphere and the ecosystem. NEE was calculated on a half‐hourly basis between 1997 and 2022, based on 20 Hz measurements of the three‐dimensional wind speed (measured by sonic anemometers; Table S1) and CO_2_ molar densities or dry mole fractions (measured by infrared gas analyzers; Table S1). Instruments were installed above the tree canopy at a height of 35 m on top of a measurement tower. Several different instrument types, that is, sonic anemometers (Gill R2: Jan 1997–Dec 2006, Gill R3‐50: Dec 2006–Nov 2016, Gill HS‐50: Jul 2014–ongoing; Gill Instruments Ltd., Lymington, Hampshire, UK) and infrared gas analyzers (LI‐6262: Jan 1997–Aug 2005, LI‐7500: Aug 2005–Nov 2016, LI‐7200: Jul 2014–ongoing; LI‐COR Inc., Lincoln, Nebraska, USA) were used during the 26‐year study period, calibrated on a regular basis (details are listed in Table S1).

Flux processing was done according to established community guidelines (Aubinet et al. 2012; Sabbatini et al. 2018). We used the EddyPro software (v6 and v7) by LI‐COR for flux calculations for all years. The flux processing steps included (1) high‐resolution raw data filtering (checks for spikes, drop‐outs, absolute limits; Vickers and Mahrt 1997), (2) two‐dimensional coordinate rotation (Wilczak et al. 2001) in combination with block averaging, (3) time lag detection (between turbulent sonic and IRGA signals) using covariance maximization, using a default time lag when no maximum was detected within the specified time window of 0–10s, and (4) correction for high‐pass (Moncrieff et al. 2004) and low‐pass filtering effects (Fratini et al. 2012; Horst and Lenschow 2009 for Li‐7200; Horst 1997 for Li‐7500). Corrections for density fluctuations (Webb et al. 1980) and IRGA self‐heating (Kittler et al. 2017) were done for the open‐path IRGA (LI‐7500) fluxes. By adding the CO_2_ storage term to the CO_2_ flux, NEE was calculated (Aubinet et al. 2001). The calculated half‐hourly fluxes were then quality‐checked by applying the following tests: (1) steady state and integral turbulence characteristics test (Mauder and Foken 2006), (2) spectral correction factor test (Sabbatini et al. 2018), and (3) IRGA signal strength/quality test. Test results were combined to an overall quality flag (0–1‐2), and fluxes with low quality (daytime fluxes: if quality flag = 2; nighttime fluxes: if quality flag = 1 or 2) were excluded from further analyses. Furthermore, fluxes were excluded (i) if they were measured under low turbulence conditions (friction velocity (u*) filtering with constant threshold = 0.29 m s^−1^), (ii) if they were identified as outliers (Hampel filter based on median absolute deviation in a running time window of 432 records = 9 days; repeated until all outliers removed), and (iii) if they were outside a physically plausible range (daytime: ±50 μmol m^−2^ s^−1^, nighttime: +30/−5 μmol m^−2^ s^−1^; nighttime range detected from highest quality nighttime fluxes). The u* filtering and the NEE time series gap‐filling using the marginal distribution sampling (MDS) method (Reichstein et al. 2005) was done in Rstudio (v1.3.959) using the R package “ReddyProc” (v1.2.2; Wutzler et al. 2018) and we aggregated the half‐hourly fluxes to daily NEE (i.e., 24‐h cumulative sums; NEE_c,DOY_) for further analyses (Figure S2). Footprint analysis was done using the Kljun et al. (2015) footprint model (Figure S3). The uncertainty in NEE was calculated as the joint uncertainty from the uncertainty estimates based on multiple thresholds for u* filtering (using the 16th and 84th percentiles, i.e., 0.19 and 0.43 m s^−1^; Pastorello et al. 2020) and from random uncertainty due to random measurement errors (Hollinger and Richardson 2005). The combination and aggregation of the uncertainty terms to annual values was done according to Pastorello et al. (2020) and the REddyProc documentation on uncertainty aggregation (Wutzler et al. 2024). The micrometeorological sign convention for EC fluxes was used, meaning that a negative NEE represents a loss of CO_2_ from the atmosphere and a net CO_2_ uptake by the forest, that is, acting as a net CO_2_ sink, while a positive NEE means that the atmosphere gained CO_2_ and the ecosystem lost CO_2_, that is, acting as a net CO_2_ source to the atmosphere. All data are openly available (Hörtnagl et al. 2023).

### 2.4 Data Analyses

#### 2.4.1 Trend Analyses

The Mann–Kendall test for monotonic trends was used to examine temporal trends in NEE and meteorological variables (Kendall 1948; Mann 1945). The slope of the Mann–Kendall trends was estimated using the Theil‐Sen method (Sen 1968). Only those trends with *p* values < 0.05 were considered significantly different from zero. Trend analysis was conducted on annual NEE (cumulative sums) and annual means of meteorological variables as well as on phenological dates (SOS and EOS) and CUP_net_ length. This was done to investigate whether there were consistent trends among environmental factors, NEE, and phenology. Moreover, we calculated the 26‐year trends of daily cumulative NEE for each calendar day of the year (DOY; NEE_c,DOY_) using the Mann–Kendall test, thus enabling the observation of NEE dynamics at higher temporal resolution (i.e., daily). To reduce noise from day‐to‐day variability, for example, due to rainy or cloudy days, we applied a 5‐day moving average to the daily NEE_c,DOY_ values for this analysis. This allowed us to study the impacts of climate change on NEE at a daily time scale and to assess trends in daily NEE specifically around the days of SOS and EOS, which could be linked to phenological shifts.

#### 2.4.2 Definition of Carbon Uptake Period

**FIGURE 1:**
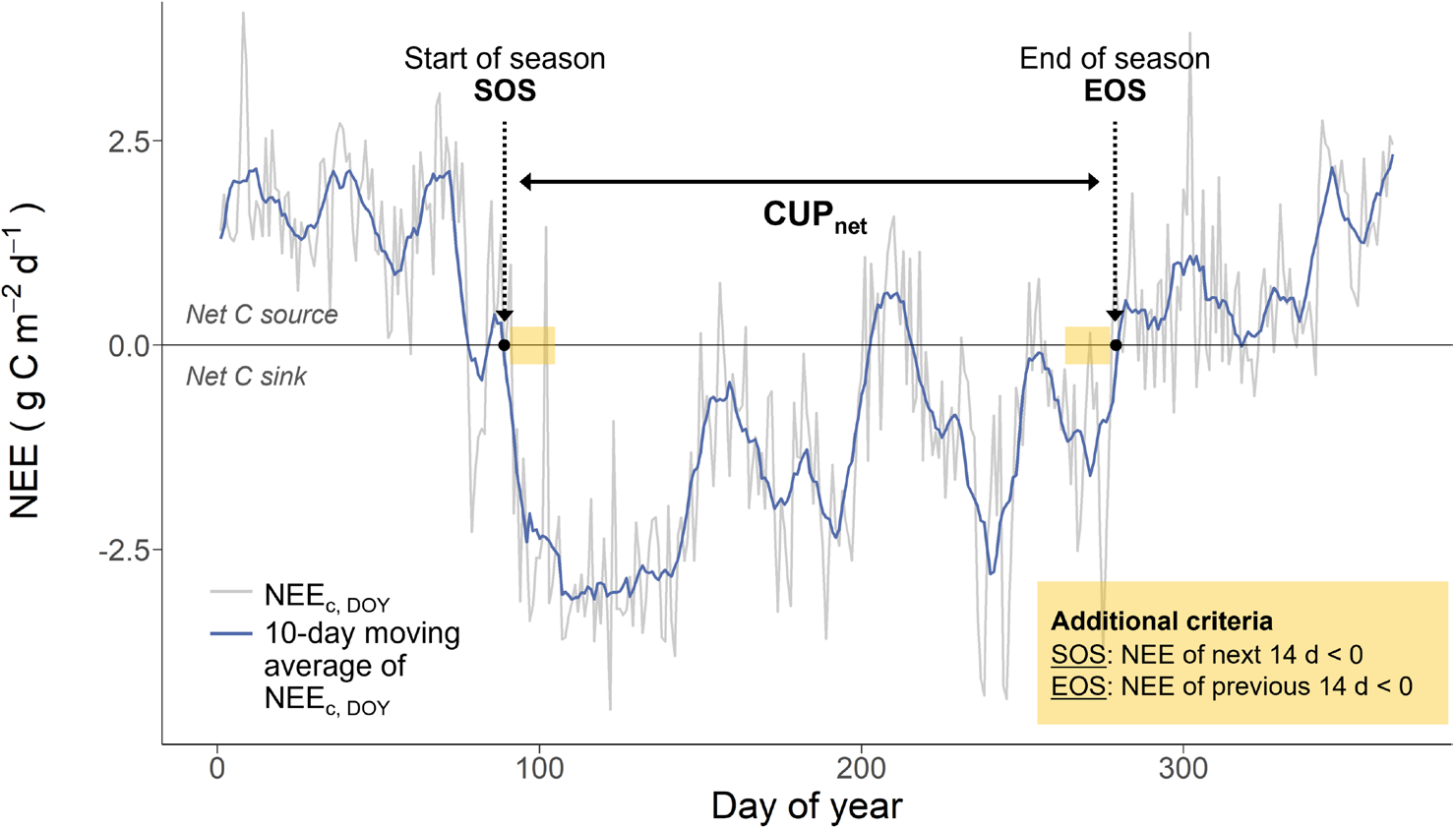
Definition of the net carbon uptake period (CUP_net_). The CUP_net_ is the period between the start of season (SOS) and the end of season (EOS), that is, the first and last zero‐crossing of the moving 10‐day average (blue line) of measured daily net ecosystem CO_2_ exchange (daily cumulative NEE; NEE_c,DOY_; grey line). We applied additional criteria for SOS and EOS: For SOS, NEE_c,DOY_ (10‐day moving average) needed to be < 0 for the following 14 d, and for EOS < 0 for the previous 14 d (marked as yellow area). Example shows the year 2018.

The CUP_net_ was determined using a 10‐day moving average of NEE_c,DOY_, following the approach of Panwar et al. (2023). The start of season (SOS) and end of season (EOS) were defined as the first and last DOY when the smoothed NEE_c,DOY_ (10‐day moving average) became negative (Figure 1). The CUP_net_ was then defined as the period between SOS and EOS, during which the smoothed NEE_c,DOY_ was consistently negative, that is, an additional criterion was applied: NEE_c,DOY_ had to remain negative for at least 14 consecutive days following SOS and 14 days preceding EOS. If these conditions were not met, the identified SOS or EOS were disregarded, and the procedure was repeated until all conditions were met. This approach helped to prevent the inclusion of single net C uptake days at the year's start or end, thereby avoiding potentially inaccurate early or late CUP_net_ dates.

#### 2.4.3 Driver Analyses With *XGBoost* and SHAP

We used an explainable machine learning approach where we combined an eXtreme Gradient Boosting (XGBoost) model, that is, a tree‐based ensemble learning model, with SHapley Additive exPlanations (SHAP) values to determine the importance of environmental drivers on NEE_c,DOY_. All data analyses were performed using R statistical software (v4.4.1; R Core Team 2024). Figures were created using scientifically derived, nondistorting, and universally readable color maps (Crameri 2020).

We trained the XGBoost model on the 26‐year NEE_c,DOY_ data set, using the “xgboost” package (v1.7.7.1; Chen et al. 2024). We selected the model features to predict NEE_c,DOY_ based on previous knowledge from the literature (Etzold et al. 2022; Körner et al. 2023; Zielis et al. 2014) and after applied principal component analysis to assess collinearity for a pre‐selection of predictor variables. The final predictor set consisted of 21 variables (Table S2), including daily mean, minimum and maximum T_air_ (T_air_mean_, T_air_min_, T_air_max_), hours of T_air_ < 0°C, Rg, PPFD, daylength, Prec, SWC, RH and VPD in their original, smoothed (using moving averages (MA) with different window sizes (*x* = 3–30), that is, an average over the previous x days), cumulative (using moving sum (MS) with different window sizes (*x* = 15–90), that is, a sum over the previous x days) and/or lagged version with different window sizes (*x* = 15–365), that is, the variable was moved forward in time. For all variables, we tested and evaluated several smoothed, cumulative or lagged versions with different window lengths and lags. We arrived at the final predictor set in an iterative process, testing several predictor sets and removing variables with low predictor importance. The smoothed, cumulative and lagged predictor versions allowed us to investigate cumulative and lagged effects of drivers on NEE_c,DOY_. For instance, the 3‐day MA of T_air_mean_ (T_air_MA3_) and the 5‐day MA of T_air_min_ (T_air_min_MA5_) were included since for trees a “sustained” passing of a threshold air temperature (e.g., > 5°C) is often considered essential for the start of the season (Gao et al. 2022; Körner et al. 2023). Similarly, the 15‐day MS of T_air< 0_ with 15‐day lag (T_air< 0_MS15_lag15_), the 30‐day MA of Rg with 180‐day lag (Rg_MA30_lag180_) and the 30‐day MA of T_air_mean_ with 180‐ and 365‐day lag (T_air___MA30_lag180_ and T_air___MA30_lag365_) were included since they were identified as important drivers of annual NEE at the Davos Seehornwald spruce forest in a study by Zielis et al. (2014) and Gu et al. (2022). Hyperparameter optimization of the XGBoost model was done by grid search, using the “caret” package and 10‐fold cross‐validation (Kuhn 2008). The hyperparameters used are listed in Table S2. Model training was done on 75% of the NEE_c,DOY_ data while model performance and generalization were assessed using the remaining 25% of the data (test data) using the Root Mean Square Error (RMSE) and the coefficient of determination (*R* ^2^). The final XGBoost model performed very well in modeling NEE_c,DOY_, with an *R* ^2^ of 0.71 and an RMSE of 0.94 g C m^−2^ d^−1^ on the test data.

We used SHAP values to interpret the output of the XGBoost model by investigating the relative importance of individual features, implementing the “SHAPforxgboost” package (v0.1.3; Liu et al. 2023). SHAP is founded upon game theory and can be utilized to evaluate the individual contributions of each feature to the prediction (Shapley 1953). The sum of the SHAP values for all features for any given prediction represents the difference between the prediction and the average prediction. Thus, the SHAP values are expressed in the unit corresponding to the predicted variable. The contribution of drivers to NEE_c,DOY_ over the course of the year was investigated as the mean over the 26‐year period (for SWC and PPFD, as the mean from 2007–2022 and 2001–2022, respectively). Moreover, we examined the main drivers of SOS and EOS to identify the factors influencing the forest's transition from a C source to a C sink during spring and vice versa during autumn. We used the SHAP values from NEE_c,DOY_ and calculated the difference between the median SHAP value per feature over 10 days preceding and 10 days following SOS and EOS (n_−10_‐n_−1_ vs. *n*_+1_‐n_+10_), respectively, to see which drivers had the largest change in their SHAP values around SOS and EOS and thus triggered the largest change in NEE_c,DOY_. Subsequently, the median SHAP value was determined over the 26‐year period, providing an overall estimate of the drivers for SOS and EOS.

## 3 Results

### 3.1 Long‐Term Trends in Environmental Conditions and NEE

Annual mean and maximum T_air_ showed a significant positive trend over the 26‐year period (*p* < 0.05; Table 1), with a total increase in mean annual T_air_ of about 1°C (slope = 0.037°C year^−1^, *p* = 0.022) and a total increase in maximum annual T_air_ of approx. 2°C in the period between 1997 and 2022 (slope = 0.078°C year^−1^, *p* = 0.029). In contrast, annual minimum T_air_ (slope = 0.1°C year^−1^) and the number of hours with T_air_ < 0°C (slope = −9 h year^−1^) did not change significantly (Table 1). At the seasonal level, summer minimum T_air_ increased significantly (slope = 0.12°C yr.^−1^, *p* = 0.013), while the number of hours with T_air_ < 0°C in autumn and summer decreased significantly (slope = −6.7 h year^−1^, *p* = 0.019) (Table 1). No significant changes in annual or seasonal mean or maximum values of VPD and mean Rg were found over the 26‐year period. SWC decreased significantly between 2007 and 2022 on the annual scale as well as in summer and autumn (*p* < 0.05; Table 1). Precipitation increased significantly in winter (*p* < 0.05; Table 1) but not on the annual scale.

**TABLE 1:** Means and trends in environmental variables and measured NEE over the 26‐year study period (1997–2022; 2007–2022 for SWC) for the full year as well as per season.

		Full year		Winter (DJF)		Spring (MAM)		Summer (JJA)		Autumn (SON)	
		Mean	Trend	Mean	Trend	Mean	Trend	Mean	Trend	Mean	Trend
T_air_mean_	°C	4.3 ± 0.7	0.037*	−3.1 ± 1.5	0.017	3.2 ± 1.0	0.024	12.0 ± 1.1	0.065**	5.0 ± 1.2	0.053
T_air_min_	°C	−17.5 ± 2.7	0.100	−17.0 ± 2.8	0.063	−12.9 ± 3.2	0.033	0.5 ± 2.0	0.12*	−10.5 ± 3.3	0.15
T_air_max_	°C	25.7 ± 1.3	0.078*	10.3 ± 1.6	0.040	20.0 ± 2.2	0.052	25.7 ± 1.3	0.078*	20.7 ± 1.6	−0.009
T < 0°C	hr	2662 ± 281	−9.0	1577 ± 179	−4.3	646 ± 129	−3.6	5 ± 10	−0.03*	434 ± 134	−6.7*
VPD	hPa	3.2 ± 0.3	0.01	1.8 ± 0.4	−0.01	3.2 ± 0.4	0.01	4.8 ± 0.8	0.03	2.9 ± 0.5	0.00
VPD_max_	hPa	25.6 ± 2.7	0.14	9.8 ± 1.7	0.04	17.7 ± 2.9	0.04	25.6 ± 2.7	0.14	17.7 ± 2.2	0.03
SWC	%	25.8 ± 3.4	−0.45*	25.8 ± 3.0	−0.37	28.1 ± 3.4	−0.15	25.3 ± 4.3	−0.53*	24.1 ± 5.4	−0.67*
Rg	W m^−2^	152 ± 5	0.18	74 ± 5	−0.2	196 ± 11	0.33	223 ± 13	0.55	114 ± 8	0.15
Prec	mm period^−1^	869 ± 195	0.54	132 ± 70	3.3*	160 ± 55	−0.25	369 ± 96	−4.2	208 ± 70	0.21
NEE	g C m^−2^ period^−1^	−130.1 ± 65.6	−7.8*	96.1 ± 48.5	−2.8	−121.7 ± 43.2	−1.3	−108.8 ± 47.6	−1.3	3.96 ± 39.5	−1.74**

*Note:* Numbers after the mean indicate standard deviations except for NEE where ± joint uncertainty is given (see Methods for details). Trends (i.e., slopes of relationships) were detected using the Mann–Kendall trend test with the Theil‐Sen method and are given per year. Trends with *p*‐values < 0.05 are marked with *, trends with *p*‐values < 0.01 are marked with **.

**FIGURE 2:**
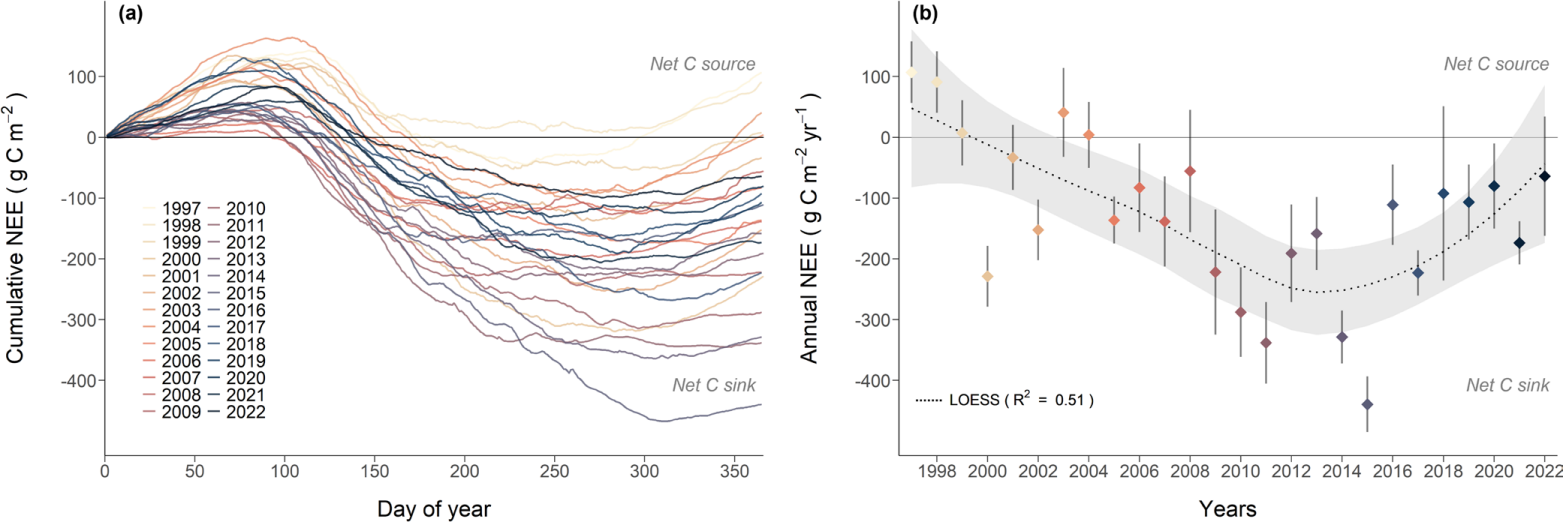
Long‐term annual NEE of the spruce forest CH‐Dav during 1997 to 2022. (a) Annual courses of cumulative NEE for 26 years and (b) annual NEE are shown. Joint uncertainty (combination of random uncertainty and u* uncertainty) is shown as error bar in panel (b). The dotted line shows the smoothed multiyear trend using a LOESS function (smoother span = 0.9; grey band = standard error). Same color code for individual years applies to both panels.

Annual cumulative NEE showed the typical intra‐annual courses with distinct seasonality in all 26 years (Figure 2a). In the beginning of any given year, that is, DOY 0–100 (January—mid‐April), NEE_c,DOY_ was predominantly positive, that is, the forest was a net C source early in the year (winter: 96 g C m^−2^; Table 1). At around DOY 100, that is, mid‐April, cumulative NEE started to decline, marking the beginning of the forest acting as a net C sink, that is, CO_2_ uptake through photosynthesis was larger than CO_2_ loss through respiration (Figure 2a). Thus, at around DOY 100–180, the forest compensated the cumulative CO_2_ loss during the beginning of the year with CO_2_ uptake, indicated by cumulative NEE crossing the zero‐line. The days with the most negative NEE_c,DOY_, that is, highest daily net CO_2_ uptake, occurred usually right at the beginning of the net C uptake season between DOY 90–150, that is, mid‐April—May. Therefore, the most productive season was spring (spring average NEE: −122 g C m^−2^; Table 1). Net CO_2_ uptake behavior (summer: −109 g C m^−2^; Table 1) occurred until around DOY 250–300, when CO_2_ losses started to exceed CO_2_ fixation (autumn: 4 g C m^−2^). In most years, cumulative NEE at the end of the year was negative, indicating that the forest was a C sink (Figure 2a). Only five out of 26 years ended with a positive annual NEE, indicating that the forest had been a net C source (1997–1999, 2003–2004). Overall, we observed a large interannual variability in the NEE, with values between −439 g C m^−2^ year^−1^ in 2015 and 107 g C m^−2^ year^−1^ in 1997. On average, the forest was a net C sink over the 26 years, with an annual NEE of −130 ± 66 g C m^−2^ year^−1^ (±joint uncertainty; see Methods for details).

Over the 26 years, we detected a significant negative trend in annual NEE (Mann–Kendall trend, *p* < 0.05; Table 1), indicating that the forest became a stronger C sink. However, the fitted LOESS curve indicated that this trend was broken; annual NEE leveled off between 2012 and 2015, followed by a decrease in the annual C sink until 2022 (Figure 2b). At the seasonal level, we found a significant trend in NEE only in autumn, which indicated that the forest's net C sink increased during autumn over the 26 years of the study (*p* < 0.01; Table 1).

### 3.2 Trends in Daily NEE Fluxes Over Time

To assess the effects of climate change on NEE throughout the study period, we analyzed the days with the strongest net C uptake, that is, 1st, 5th percentiles of NEE_c,DOY_, and strongest net C loss, that is, 95th and 99th percentiles of NEE_c,DOY_, as well as the median NEE_c,DOY_ during the 26‐year period (Figure S4). We observed that the 95th and 99th percentiles of NEE_c,DOY_ significantly decreased (slopes = −0.04 and −0.05 g C m^−2^ year^−1^, respectively, *p* < 0.05), that is, indicating a reduced C source on peak net C source days (Figure S4). For the 1st and 5th percentiles of NEE_c,DOY_, we did not find any significant trends, that is, the strength of peak net C sink days did not change over the 26 years (Figure S4). We did not find a significant trend for median NEE_c,DOY_.

**FIGURE 3:**
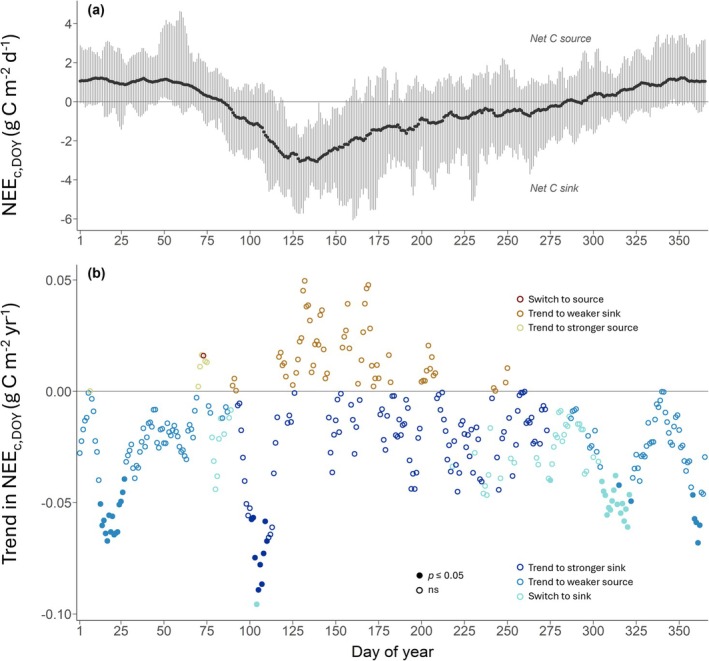
Means and trends of daily cumulative NEE (NEE_c,DOY_) of the spruce forest CH‐Dav during 1997 to 2022. (a) 5‐day moving averages of NEE_c,DOY_, averaged over 26 years, are shown. Grey bars depict minimum and maximum values of NEE_c,DOY_ over the period 1997–2022. (b) Slopes of the trends in NEE_c,DOY_ over the period 1997–2022, calculated for 5‐day moving averages of NEE_c,DOY_ using the Mann–Kendall test and the Theil‐Sen estimator (colored, filled circles with *p* < 0.05; colored, open circles with *p* > 0.05). Yellow circles depict days of the year (DOYs) with positive NEE_c,DOY_ and positive trends, that is, C source getting larger. Orange circles depict DOYs with negative NEE_c,DOY_ and positive trends, that is, C sink getting smaller. Red circles depict DOYs with a switch from negative to positive NEE_c,DOY_, that is, a switch from C sink to C source. Dark blue circles depict negative NEE_c,DOY_ and negative trends, that is, C sink getting larger. Blue circles depict DOYs with positive NEE_c,DOY_ and negative trends, that is, C source getting smaller. Turquoise circles depict DOYs with a switch from positive to negative NEE_c,DOY_, that is, a switch from C source to C sink. See Figure S5 for a visualization of the six trend categories.

To determine whether changes in annual NEE were due to changes in daily NEE and/or shifts in C phenology (see Section 3.3), we first investigated NEE_c,DOY_, averaged over 26 years. We found large intra‐annual, short‐term variations in mean NEE_c,DOY_, reflecting the typical annual course of forest CO_2_ fluxes (Figure 3a). Net CO_2_ losses prevailed early in the year (January, February; positive mean NEE_c,DOY_), net CO_2_ uptake was largest in spring (MAM; negative mean NEE_c,DOY_) and smaller in the summer months (JJA). During autumn (SON), small net CO_2_ uptake and small net CO_2_ losses were observed (NEE_c,DOY_ fluctuated around the zero‐line), before net CO_2_ losses dominated again in December (positive NEE_c,DOY_).

We then investigated changes in NEE_c,DOY_ during the period 1997–2022, since we expected negative trends in NEE_c,DOY_ during spring and autumn, that is, increasing net CO_2_ uptake or decreasing net CO_2_ losses. Indeed, we found 48 days with significant negative trends in NEE_c,DOY_, indicating that the forest had higher net CO_2_ uptake (acted as a larger C sink) or had lower net CO_2_ losses (acted as a smaller C source) on these 48 days (Figure 3b; filled blue circles below the zero line). Especially in winter (DJF; DOY 355–365 and DOY 1–40), we found clusters of days with negative trends in NEE_c,DOY_ (on 21 days in total; *p* < 0.05), clearly reflecting that the forest became a smaller net C source on these days (blue filled circles in Figure 3b). Additionally, the forest switched from being a net C source to being a net C sink (negative trends in NEE_c,DOY_ crossing the 0‐line; *p* < 0.05) on 1 day in spring (DOY 104, mid‐Apr) and on 17 days in autumn (Oct–Nov, DOY 275–325; turquoise filled circles in Figure 3b). Moreover, on 9 days, the forest became a stronger net C sink in spring (negative trends in NEE_c,DOY_; *p* < 0.05; dark blue filled circles in Figure 3). In contrast, we did not detect any significant negative trends in summer (i.e., forest becoming larger net C sink) nor any significant positive trends in NEE_c,DOY_ (i.e., forest becoming larger net C source or switching to net C source; yellow and red circles in Figure 3).

### 3.3 Net Carbon Uptake Period (CUP_net_ ) Over Time

**FIGURE 4:**
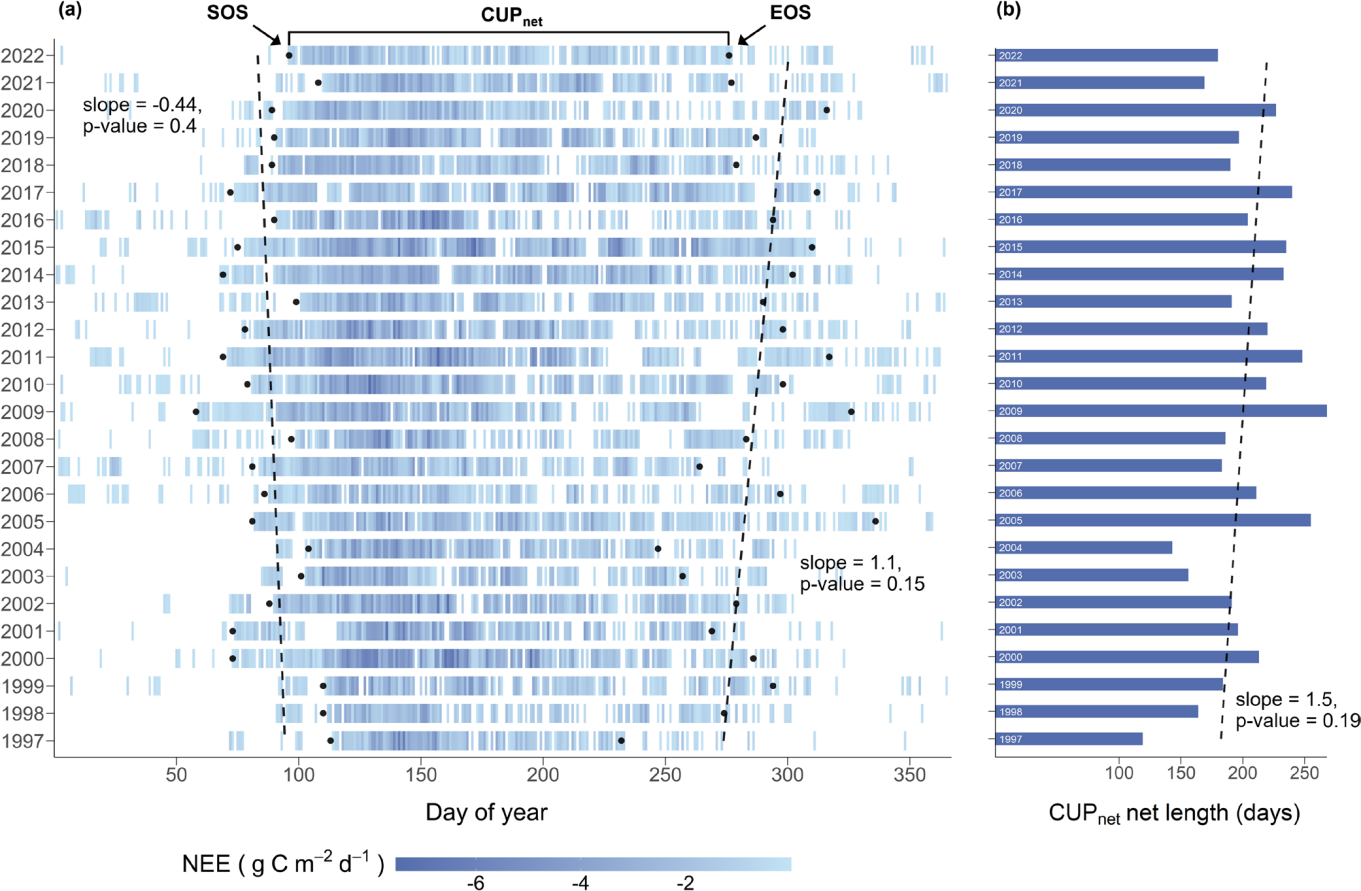
Net carbon uptake period (CUP_net_) of the CH‐Dav spruce forest for years between 1997 and 2022. (a) 10‐day averages of daily NEE were used to identify CUP_net_, additional criteria were used to identify start and end of season (SOS and EOS, respectively; see text and Figure 1 for further details). Color scale for daily NEE is based on measured NEE, representing daily net uptake strength of the forest (negative NEE, i.e., C sink only). Black dots represent SOS and EOS. Black dashed lines show Mann–Kendall trends for SOS and EOS over 26 years (both insignificant, *p* value > 0.05). (b) CUP_net_ length per year is shown. Mann–Kendall trend (black dashed line) was insignificant over 26 years (*p* value > 0.05).

We assessed potential shifts in phenology at the Davos spruce forest based on the net carbon uptake period (CUP_net_). The start of season (SOS) was typically around DOY 88 (late March) and varied by ±15 days during the 26 years, while the end of season (EOS) was around DOY 288 (mid‐October) and varied by ±24 days (Figure 4a). The earliest SOS was on DOY 58 (late February) in 2009, while the latest was on DOY 113 (mid‐April) in 1997. The earliest EOS was on DOY 232 (mid‐August) in 1997, and the latest was on DOY 336 (early December) in 2005. Over the entire 26 years, SOS started slightly earlier (−0.4 days year^−1^) and EOS slightly later (+1.1 days year^−1^); however, these shifts were not significant (*p* > 0.05; Figure 4a). Interestingly, this insignificant trend for EOS changed in 2009: we found a significant trend for earlier (not later) EOS between 2009 and 2022 (using change point detection, *p* = 0.032). Moreover, CUP_net_ length was on average 201 days and showed a high variability among years (SD: ±35 days; Figure 4b). Thus, we did not observe any significant trend, although CUP_net_ length tended to increase over time (+1.5 days year^−1^; *p* > 0.05). The longest CUP_net_ was in 2009 with 268 days, and the shortest CUP_net_ was in 1997 with 119 d. Overall, we did not find clear signs of phenological shifts like an earlier start and/or delayed end of the active season at our study site over the course of 26 years of our study.

**FIGURE 5:**
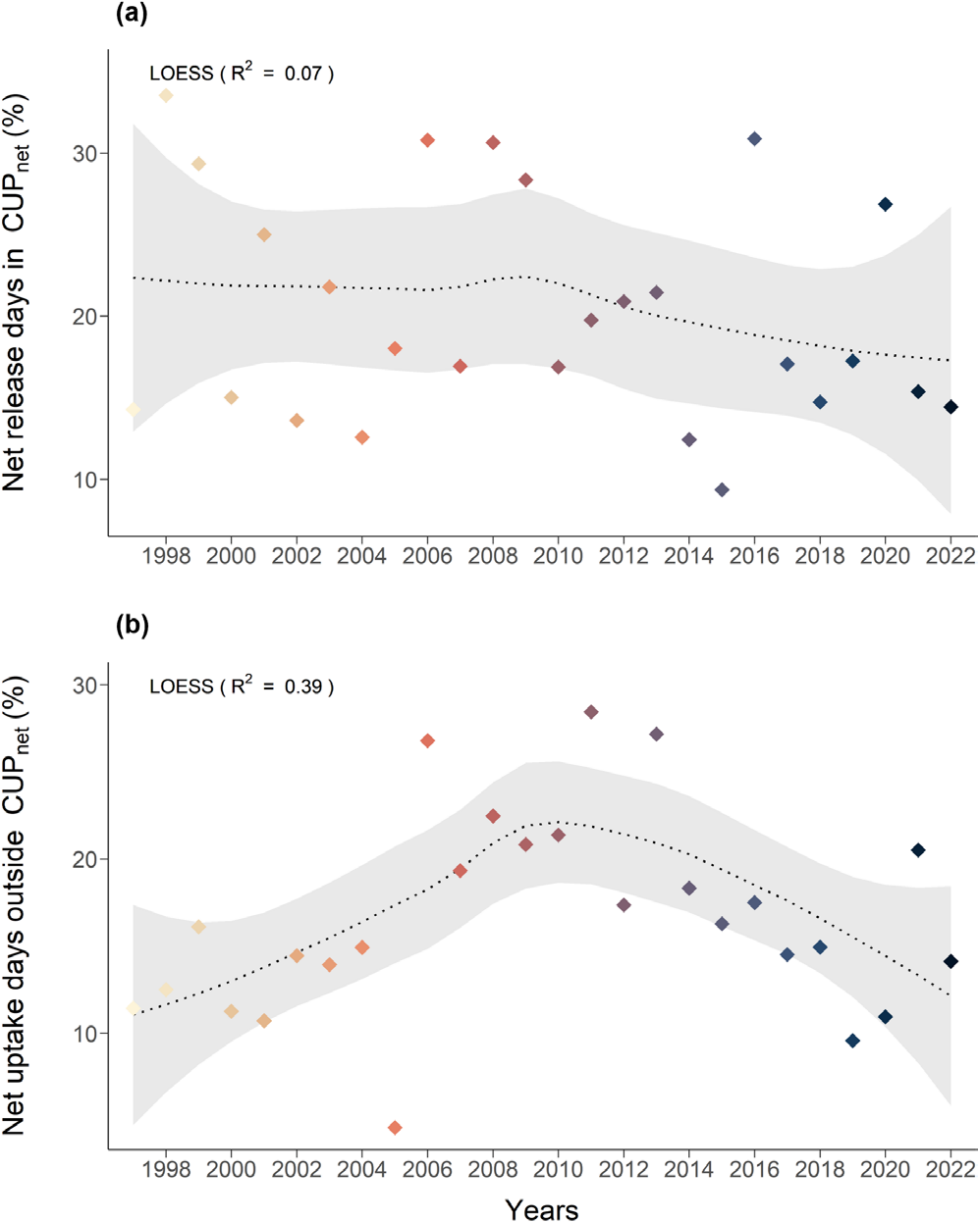
Net release days within CUP_net_ and net uptake days outside CUP_net_ for years between 1997 and 2022. (a) percentage of days within CUP_net_ showing net CO_2_ release, that is, positive daily cumulative NEE (NEE_c,DOY_) and (b) percentage of days outside of CUP_net_ showing net CO_2_ uptake, that is, negative NEE_c,DOY_. The dotted line shows the smoothed multiyear trend using a LOESS function (smoother span = 0.9; grey band = standard error).

During each CUP_net_, there were 17–76 days when the forest emitted CO_2_ (Figure 4a), with the proportion of these net release days within the CUP_net_ relative to the total CUP_net_ gradually declining over the 26‐year study period (Figure 5a). On the other hand, we observed 5–47 net C uptake days outside the CUP_net_, with their proportion increasing from 1997 to around 2013, peaking at ~25% in 2009, before decreasing to ~15% in 2022 (Figure 4a, Figure 5b).

**FIGURE 6:**
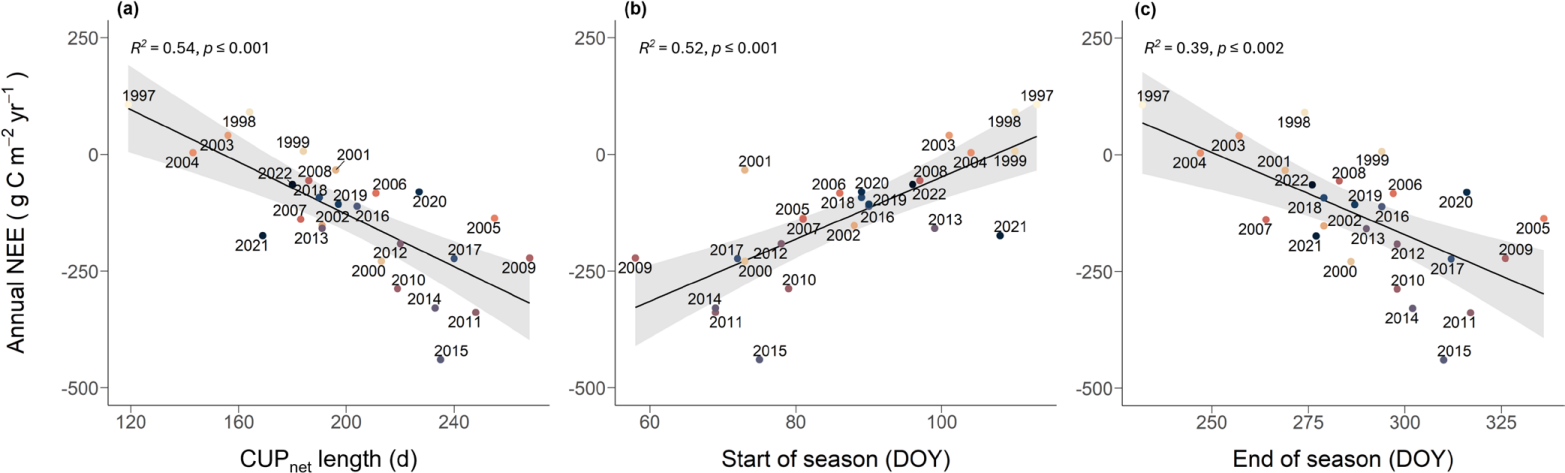
Relationships between annual NEE and phenology for the spruce forest CH‐Dav during the period 1997–2022. Cumulative annual NEE in relation to (a) net carbon uptake period (CUP_net_) length, (b) DOY of start of season (SOS) and c) DOY of end of season (EOS). Linear regressions with 95% confidence bands are shown (all: *p* ≤ 0.002).

Despite no clear trends in carbon phenology dates over time, we observed that the longer the CUP_net_ length, the larger the annual total C sink (*R* ^2^ = 0.54; *p* < 0.001; Spearman rank correlation coefficient *ρ* = −0.73; Figure 6a). Moreover, an earlier SOS (*R* ^2^ = 0.52, *p* < 0.001; Figure 6b) and a later EOS (*R* ^2^ = 0.39; *p* < 0.01; Figure 6c) coincided with higher annual net C sinks of the forest.

### 3.4 Contribution of Drivers to NEE Dynamics at Daily Time Scales

**FIGURE 7:**
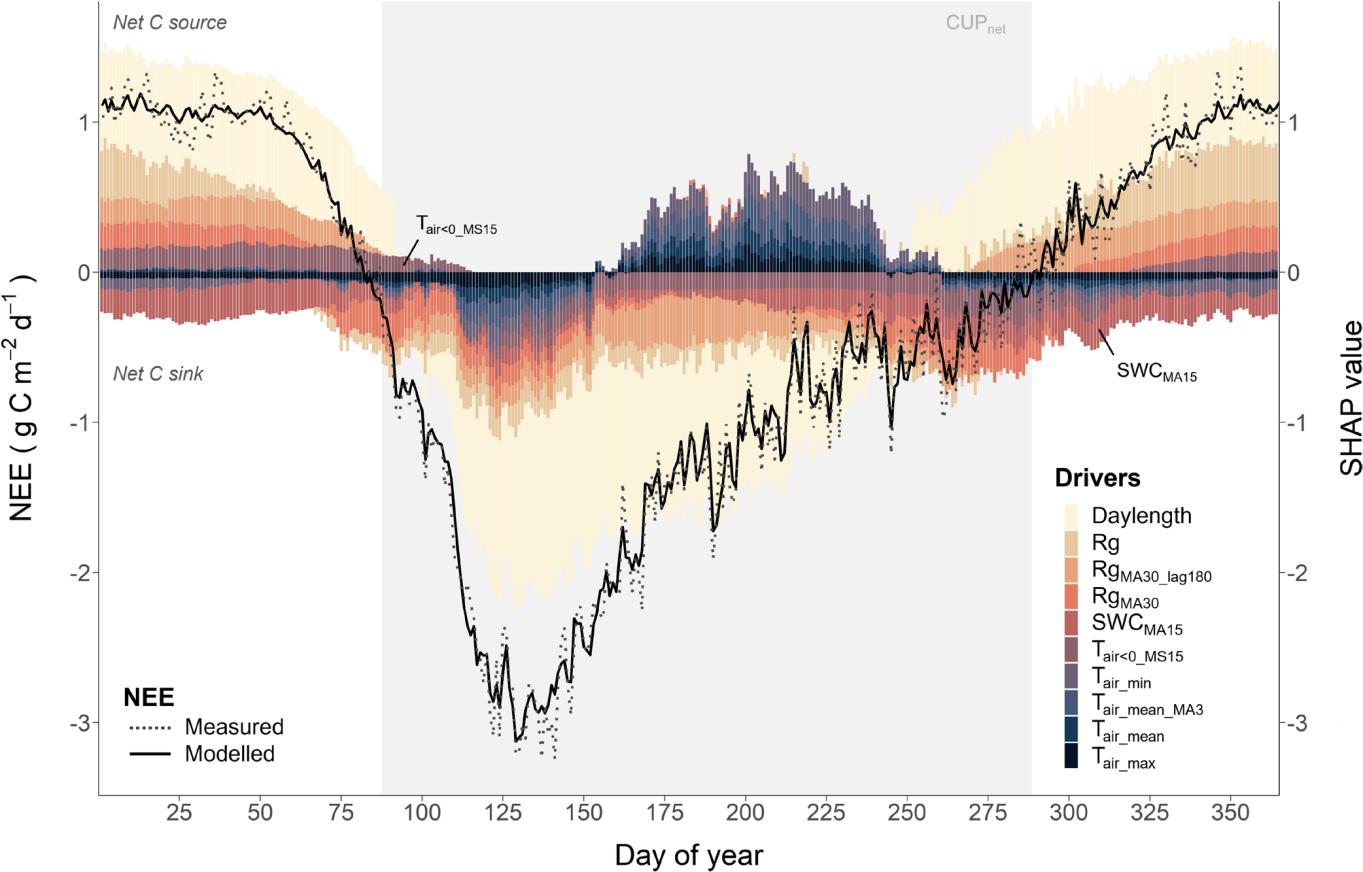
Driver analysis for mean daily cumulative NEE (NEE_c,DOY_) of the spruce forest CH‐Dav during the period 1997–2022. Mean daily SHAP values of the ten most important XGBoost model features to predict NEE_c,DOY_ over 26 years are given (stacked colored bars; see Table S2 for abbreviations). The mean model prediction was −0.35 g C m^−2^ d^−1^, for which the sum of all SHAP values is 0. Positive SHAP values impact modeled NEE toward more positive NEE (i.e., stronger C source or weaker C sink), while negative SHAP values impact modeled NEE toward more negative NEE (i.e., weaker C source or stronger C sink). The 26‐year NEE_c,DOY_ is shown as the grey dotted line (based on measured data), while the modeled 26‐year mean NEE_c,DOY_ is shown as the black solid line. The shaded area represents the mean net carbon uptake period (CUP_net_).

The XGBoost model combined with the SHAP analysis allowed the daily attribution of drivers to explain NEE_c,DOY_ dynamics, resulting in unprecedented insights into the responses of the spruce ecosystem to its environment over the 26 years of the study (Figure 7). The XGBoost model performed very well (see Methods) and captured the seasonal dynamics, including the zero‐crossing dates for the 26‐year flux time series (Figure 7; black dotted and solid lines). NEE_c,DOY_ was strongly related to daylength and Rg (immediate Rg as well as lagged Rg_MA30_ and Rg_MA30_lag180_), increasing the net C loss during autumn and winter (positive SHAP values) and increasing the net C uptake during spring and summer (negative SHAP values). Furthermore, the 15‐day moving average of SWC (SWC_MA15_) and the number of hours per day with T_air_ < 0°C cumulated over the 15 previous days (T_air< 0MS15_) were important drivers of NEE_c,DOY_. SWC typically increased the net C uptake (negative SHAP values throughout the year; Figures 7, S6), whereas a low prevalence of T_air_ < 0°C increased the probability for net C uptake (Figure S6; positive SHAP values in winter). Air temperature‐related drivers, that is, T_air_min_, T_air_mean_, T_air_max_, and T_air_mean_MA3_ were the next most important variables, all showing nonmonotonic relationships with NEE_c,DOY_ (Figure S6; see below). VPD was found to be the least important driver.

In general, we found a pronounced seasonality in driver contributions to explain NEE_c,DOY_, as indicated by the SHAP values for almost all drivers (Figure 7; except for SWC, Figure S6). Drivers changed their effect (indicated by the sign of SHAP values) from increasing the net C loss to increasing the net C uptake in spring (shift from positive to negative SHAP values) and vice versa in autumn, thereby strongly driving the seasonality of NEE_c,DOY_. The timing of this shift in contributions of almost all drivers coincided well with the mean CUP_net_ identified for the 26 years of study (Figure 7; grey area). All drivers contributed to an increase in net C uptake (negative SHAP values) in late spring (DOY 100–150; MAM), in accordance with most negative NEE values (Figures 3, 7; Table 1).

**FIGURE 8:**
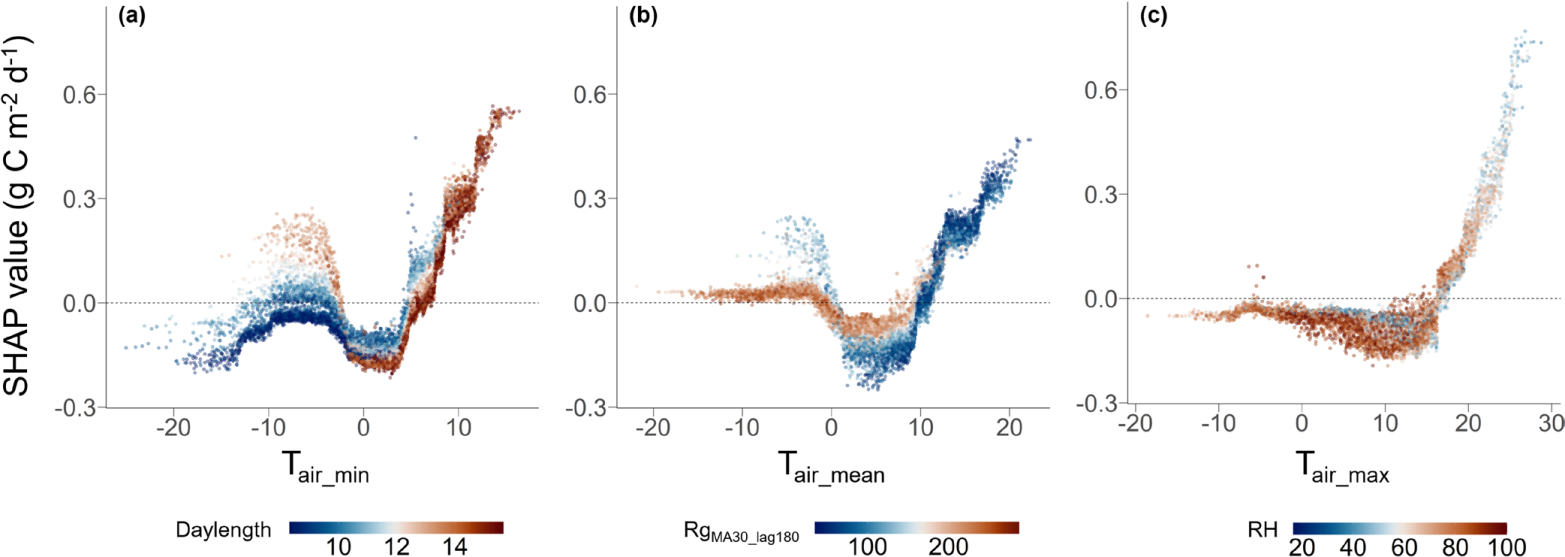
Partial dependence plots of SHAP values predicting daily NEE of CH‐Dav over 26 years. SHAP values for (a) minimum, (b) mean, and (c) maximum daily air temperature (T_air_min_, T_air_mean_ and T_air_max_) are given. Colors represent feature values of the strongest interacting feature (i.e., daylength in hour); 30‐day moving average of short‐wave incoming radiation with 180‐day lag (Rg_MA30_lag180_); mean relative humidity (in hPa; RH). See Table S2 for abbreviations.

Interestingly, drivers connected to temperature (T_air_mean_, T_air_max_, and T_air_min_) increased the net C uptake (indicated by negative SHAP values) in spring (DOY100–150) and autumn (DOY250–300), while these same drivers limited the net C uptake in winter and especially in summer, suggesting that high temperatures in summer strongly limited the net C uptake of the forest (positive SHAP values; Figure 7). The most favorable temperatures for C uptake (Figure 8) were: T_air_min_ between −2.5°C and 4.5°C (SHAP values for T_air_min_ became positive below −2.5°C or when exceeding 4.5°C), mean T_air_ < 11.5°C (positive SHAP values for T_air_mean_ when exceeding 10°C–11.5°C), and T_air_max_ < 17.5°C (positive SHAP values for T_air_max_ exceeding 14.5°C–17.5°C). Interestingly, the strongest interacting feature of T_air_max_ in the model was relative humidity, suggesting a link to VPD being a limiting factor under high temperatures. Furthermore, mean SHAP values of all temperature‐related drivers in summer (JJA) had significant positive trends over the 26 years (*p* < 0.05; Figure S7), suggesting that the limiting effect of T_air_ on net C uptake became stronger over the 26 years, probably driven by the strongly rising summer temperatures (+2°C, 1997–2022; Table 1).

To better understand which of the drivers of NEE_c,DOY_ changed the most in their impact on NEE_c,DOY_ around SOS and EOS, we compared SHAP values 10 days before versus 10 days after those dates for the 26 years of study. Daylength showed the largest change in SHAP values (Table 2), driving the switch from net C loss to net C uptake of the spruce forest in spring (when daylength was most important for NEE_c,DOY_) and back to net C loss in autumn (Table 2). For SOS, minimum T_air_ and 5‐day moving average of T_air_min_ were the next most important drivers. In contrast, for EOS, radiation was the next most important driver for the shift in NEE_c,DOY_ from net C uptake to net C loss.

**TABLE 2:** Difference in median SHAP values during 10 days before versus 10 days after the respective annual SOS and EOS date for the eight most important model features driving SOS and EOS. The two most important features are highlighted in bold. Negative values indicate that the SHAP values of a specific model feature decreased after SOS/EOS respective to before, while positive values indicate that the SHAP values increased. See Table S2 for abbreviations.

	Daylength	Rg	Rg_MA30_lag180_	Rg_MA30_	T_air_min_	T_air_mean_	T_air_mean_MA3_	T_air_max_
SOS	**−0.315**	−0.034	−0.086	−0.010	**−0.158**	−0.069	−0.098	−0.018
EOS	**0.066**	**0.048**	0.024	0.034	0.014	0.007	0.040	0.010

## 4 Discussion

### 4.1 Climate Change Effects on NEE of a Norway Spruce Forest

Switzerland—like the rest of Europe–has experienced climate change‐induced increases in T_air_ (CH2018 2018) and VPD (Shekhar, Buchmann, et al. 2024), and more frequent and intense droughts and heatwaves have occurred in the last decades (Scherrer et al. 2016, 2022). Our subalpine site is no exception: over the last 26 years, we observed increases in mean annual T_air_ of ~1°C and in mean annual maximum T_air_ of ~2°C, significant decreases in soil moisture, but no significant changes in annual precipitation sums (Table 1), leading to pronounced effects on forest performance. The observed nonlinear trends in annual NEE (Figure 2) reflect the complexity of ecosystem responses to these changing environmental conditions, which are shaped by nonlinear processes and seasonal variations. Norway spruce, a cold‐adapted boreal mountainous tree species, has been reported as strongly sensitive to high temperatures and summer drought (Ammer et al. 2008; Martes et al. 2024), indeed one of the most sensitive species in Europe to dry and warm conditions (Martes et al. 2024). The bioclimatic envelope for spruce is characterized by relatively low mean T_air_ (−4.5°C–9.5°C, Ammer et al. 2008; −6.9°C–12.8°C, Martes et al. 2024), a wide range of annual precipitation (375–1200 mm; Ammer et al. 2008), and maximum T_air_ of the warmest month of 17.3°C–31.1°C as lower and upper ranges for spruce distribution (Martes et al. 2024). At our subalpine site, the temperature range supporting a net C sink of the Norway spruce forest was small, with daily mean T_air_ between −2.5°C and 11.5°C, and maximum T_air_ of 17.5°C (Figure S6). Thus, based on climatology, the Davos forest still grows within the natural temperature distribution range of Norway spruce.

Over the 26 years with NEE measurements at Davos, the spruce forest was typically a C sink, with an annual NEE of −130 ± 66 g C m^−2^ year^−1^ (Table 1, Figure 2b). This net C sink of CH‐Dav was within the range of sink strengths of other evergreen needle‐leaf forests across Europe (Foken et al. 2021; Gharun et al. 2025; Martínez‐García et al. 2024). We observed a weakening of the C sink between 2012 and 2015 and further decreases after 2015, supporting reports of declining forest C sinks in Switzerland since 2010 (FOEN 2024), in Germany since 2017 (BMEL 2024), across Europe (Korosuo et al. 2023) as well as for forests in the Northern hemisphere forests since 2015 (Ke et al. 2024). Many reasons were given to explain these trends, including increased harvest intensities, wildfires, windstorms, adverse weather conditions, extreme events like droughts and heatwaves reducing net CO_2_ uptake and/or increasing net CO_2_ loss, often followed by strong bark beetle infestations, leading to increased tree mortality. At our forest site, all these factors can be excluded as potential explanations except for extreme weather events, which did indeed happen and have been shown to reduce net CO_2_ uptake at our site in a previous study (Shekhar, Hörtnagl, et al. 2024). Moreover, evergreen needle‐leaf forests like Norway spruce forests were shown to be particularly vulnerable to atmospheric dryness compared to deciduous and evergreen broad‐leaf, as well as mixed forests (Shekhar et al. 2023). Thus, the projected increase in the occurrence of extreme events will likely have strong negative consequences on the capacity of evergreen needle‐leaf forests, as the one at Davos, to act as reliable nature‐based solutions against climate change in the future.

### 4.2 Carbon Flux Phenology

With harvests and mortality from sudden disturbances excluded as explanations for the variability in NEE of the Davos spruce forest, changes in carbon uptake phenology were studied in detail. Over the 26 years, we found a net carbon uptake period (CUP_net_) of 201 ± 35 days (Figure 4), the season typically started around DOY 88 (late March; SOS, ±15 days) and ended around DOY 288 (mid‐October; EOS, ±24 days). For Norway spruce in the Swiss Alps, Vitasse et al. (2021) reported an advance of SOS in leaf‐out by −0.28 and −2.88 days per decade, consistent with the advance of 1–3 days per decade reported by Rahmati et al. (2023) for European forests. At our site, due to large interannual variabilities, changes in SOS, EOS, and CUP_net_ over time (−0.4 days year^−1^, +1.1 days year^−1^, +1.5 days year^−1^, respectively) were not significant, although there was a significant trend toward CUP_net_ ending earlier after 2009. Earlier EOS dates with less CO_2_ uptake due to autumn warming were already observed for northern forests (Piao et al. 2008) and recently for European forests due to increased VPD (Rahmati et al. 2023). As in other studies (Piao et al. 2019; Rahmati et al. 2023), EOS of the Davos forest was more variable than SOS (±24 vs. ±15 days). At our site, daylength played a dominant role for both SOS and EOS (Table 2), but SOS was also driven by minimum T_air_ and EOS by radiation, suggesting a link to ecophysiological processes. CO_2_ uptake and thus growth are known to increase with warmer temperatures, for example, overcoming chilling requirements (affecting SOS; Wang et al. 2020, 2022), while both CO_2_ uptake and growth slow with decreasing radiation (affecting EOS; Schulze et al. 2019). The dominance of processes related to CO_2_ uptake and not to CO_2_ release was also described by Yin et al. (2020), particularly for evergreen forests where detection of seasonality is inherently more difficult than for deciduous forests (Gamon et al. 2016; Korpela et al. 2023; Wu et al. 2017). While models incorporating T_air_ and daylength successfully simulate SOS and EOS in many cases (e.g., Bowling et al. 2024), challenges remain in fully capturing the complexity of phenological processes, particularly in evergreen forests, due to interactions with other factors such as radiation, soil water status, VPD, as well as site‐specific differences in temperature sensitivity of phenology (i.e., differences in chilling requirements, temperature thresholds; Keenan et al. 2014; Piao et al. 2019; Yang et al. 2024).

We found significant relationships of annual NEE with CUP_net_, SOS, and EOS over the 26 years of study (Figure 6). Longer CUP_net_, earlier SOS, and later EOS were associated with a larger net CO_2_ sink, despite no significant temporal trends in these phenological metrics. However, only around 50% of the variation in annual NEE was explained by SOS, EOS, and CUP_net_ (Figure 6b), reflecting that the potential for photosynthesis does not guarantee high GPP nor strong net CO_2_ uptake. Other factors, such as environmental conditions during and outside the CUP_net_, also play a major role in driving annual NEE. For example, between 10% and 25% of the days outside the CUP_net_ showed net CO_2_ uptake (Figure 5), similar to a subalpine coniferous forest in the Rocky Mountains of Colorado, USA (Monson et al. 2005) and a deciduous broadleaf forest in France (van der Woude et al. 2023). This number of net CO_2_ uptake days outside the CUP_net_ showed a pronounced temporal pattern, decreasing after 2009 (Figure 5b), coinciding with an earlier EOS. Together, these changes contributed to an increase in annual NEE (weaker C sink) in the more recent years (Figure 2b). Thus, the number of days with favorable conditions within the CUP_net_ seems responsible for the observed patterns of phenology with annual NEE, and not a uni‐directional change of phenology over time, supporting our conclusion that the Norway spruce forest is –at the annual scale– currently still well adapted to prevailing climatic conditions.

Although ecosystem carbon phenology like CUP_net_, based on EC fluxes, is not identical to leaf phenology (i.e., leaf‐out to leaf fall) based on ground and remote sensing observations or wood phenology based on stem diameter changes or tree rings, comparing phenology across scales might help constrain the underlying processes linking phenology and daily net CO_2_ uptake at the ecosystem scale. A previous study at the site by Etzold et al. (2022) found that stem growth occurred discontinuously during the growing season with trees growing only during about 50% of their “window of opportunity”, supporting our observation of net CO_2_ release days during the CUP_net_. The authors concluded that annual stem growth of Norway spruce at the site was rather determined by the number of growing days per year with favorable conditions (i.e., low VPD, high soil water availability and long daylength) than by the length of the growing season, supporting our conclusion that annual NEE is rather determined by daily NEE rates in response to environmental conditions than by CUP_net_ length. Moreover, ecosystem‐level CUP_net_ length (200 days, from late March to mid‐October; Figure 4) was substantially longer than the median stem growth window at our site (110 days, from early June to late September; Zweifel et al. 2024). This difference was particularly driven by SOS, which occurred about 70 days earlier for CUP_net_ (DOY 88) compared to the start of stem growth (DOY 158), while EOS was only around 20 days later than the end of stem growth (DOY 288 vs. DOY 268, respectively). Highest stem growth rates occurred in early July (DOY 184), whereas peak NEE_c,DOY_ occurred earlier, between early May and early June (DOY 130–160). These findings suggest that early in the season, when the forest exhibited the strongest C sink (DOY 100–165), CO_2_ uptake is not invested into stem growth but rather into repair and/or development of new shoots and roots (Bergh et al. 1998). Webcam images from our site confirmed that new needles grew before DOY 138–145 (data not shown). In the 20 days of net CO_2_ sink after the end of stem growth (i.e., late September to mid‐October), trees seemed to invest in carbohydrate storage rather than stem growth. Due to the shorter and later window of stem growth compared to CUP_net_, stem growth in the future is likely to be more susceptible to extreme environmental conditions like drought or heatwaves compared to annual NEE, because such conditions occurred more frequently in summer (July–September, peak in August) than in spring (Shekhar, Hörtnagl, et al. 2024). Overall, these results suggest that climate‐driven phenology across scales was relevant, but ecosystem responses to environmental conditions dominated inter and intraannual variations of daily NEE.

### 4.3 NEE Responses to Environmental Drivers

Analyzing the main environmental drivers of daily NEE over the last decades, that is, daylength and radiation, SWC and T_air_, confirmed that they were highly seasonal (Figure 7) and mainly consistent with those of tree growth (Etzold et al. 2022). Daylength was identified as one of the most important controlling factors for daily NEE (Figure 7) and phenology (CUP_net_, SOS, EOS; Table 2), consistent with previous research (Körner and Basler 2010). However, since daylength does not change over time, it only explains intra‐annual variability in NEE, but not interannual trends. Nevertheless, it sets the baseline for other factors like T_air_ and Rg, driving photosynthesis and respiration, and thus daily NEE.

T_air_ emerged as a complex driver with its impacts varying seasonally: increasing net CO_2_ uptake in spring, autumn and winter (Figure 3), but decreasing the net CO_2_ uptake in summer (Figure 7). Annual T_air_ increased over time (Table 1) but mostly stayed within the favorable range for Norway spruce, extending the so‐called “window of opportunity” for productivity in temperature‐limited ecosystems (Fu et al. 2023; Körner et al. 2023). This response to (increasing) T_air_ was particularly relevant in spring, when daily NEE was highest, and higher temperature promoted earlier and stronger net CO_2_ uptake. Analyzing a shorter time series of NEE for the CH‐Dav site (1997–2011) than in the present study (1997–2022), Zielis et al. (2014) had already demonstrated that spring NEE was decisive for the annual C sink, accounting for about 56% of annual NEE. Interestingly, the relevance of spring temperature for NEE and tree growth increased during the past century: at the same site, Churakova et al. (2014) found that spring T_air_ explained 1% of spruce tree ring width variation from 1876 to 2006, 8% from 1950 to 2006, and 47% from 1997 to 2006. In contrast, summer temperatures had strong unfavorable effects on daily net CO_2_ uptake of the forest over the 26 years of our study (Figures 7, S7). A combination of higher soil respiration (Krebs et al. 2024), increased plant respiration and constrained acclimation of photosynthesis to warming (Crous et al. 2022), lower canopy conductance due to down‐regulation of needle stomatal conductance (Gharun et al. 2020), discontinuous tree growth (Etzold et al. 2022), and increased litterfall can explain the reduced net CO_2_ uptake in response to high summer temperatures. Thus, overshooting the optimum temperature range for net C sinks can potentially interrupt the “window of opportunity”, as observed in boreal ecosystems (Wang et al. 2018).

CO_2_ uptake rates can be even more limited when high temperatures coincide with low water availability (Shekhar, Hörtnagl, et al. 2024; Štroch et al. 2010), because spruce trees' isohydric behavior leads to reduced photosynthesis rates under low soil water availability (Vitali et al. 2017; Wiley et al. 2018). Extreme dryness conditions were observed at the Davos site between 2007 and 2022 (Shekhar, Hörtnagl, et al. 2024), with strong negative effects on the net C sink, which were mainly driven by atmospheric dryness (via VPD) and not soil dryness (via SWC) during these extreme conditions. Considering all 26 years, SWC did not limit the Norway spruce forest at Davos, despite having decreased over time (Table 1), particularly in summer and autumn. It was the only driver consistently increasing daily net CO_2_ uptake on average during the 26 years of the study (Figures 7, S6). Moreover, extreme soil drought conditions typically occurred in August and September (Shekhar, Hörtnagl, et al. 2024), not during peak net CO_2_ uptake months (Figures 3, 7, Table 1). Globally, a negative soil moisture trend was observed (Zhang et al. 2024), and global vegetation showed increased sensitivity to soil moisture (Li et al. 2022); this sensitivity was present mainly in arid and semiarid regions, strongly modulated by LAI. At our site, LAI was around 4 and rather constant during the 26 years of the study, supporting our findings that SWC was not one of the main drivers.

VPD had previously been found to be a primary driver for daily stem growth of Norway spruce at our site (1997–2022; Etzold et al. 2022), and as one of the key drivers describing forest NEE dynamics under extreme conditions (SWC, VPD, LAI and forest type, 2007–2022; Shekhar et al. 2023), suggesting that very high VPD values limit photosynthesis and tree growth (Schulze et al. 2019) and reduce net CO_2_ uptake under extreme conditions. However, our findings showed that VPD was the least important driver affecting daily NEE, probably because periods with extreme VPD values accounted for only about 10% of the respective growing seasons at Davos (Shekhar, Hörtnagl, et al. 2024), not long enough to explain intra‐ and interseasonal NEE variations over 26 years. Nevertheless, we found that the most important interacting factor for maximum T_air_ in the XGBoost model was relative humidity, indicating that high temperatures are particularly limiting for net CO_2_ uptake when coinciding with low relative humidity, providing a link to VPD. However, how changes in water supply will affect evapotranspiration (Yang et al. 2023; Zhang et al. 2016) and water‐use efficiency of the spruce ecosystem (Gharun et al. 2020, 2021) remains to be seen.

Overall, over the past 26 years, the Davos spruce forest has benefitted more from warmer spring and autumn temperatures than it has been limited by summer temperatures (Figure 3), despite the weakening C sink strength in the last years. However, increasing VPD, decreasing SWC, and further increasing temperatures are expected to negatively impact the net C sink strength of this subalpine spruce forest in the future.

## 5 Conclusions

Our study demonstrated that the subalpine spruce forest in Switzerland has acted as a net C sink between 1997 and 2022, contributing to climate change mitigation during this period. However, the forest's C sink strength has weakened in recent years. While temperature‐limited ecosystems, like our high‐elevation Norway spruce forest, may initially benefit from increased productivity in autumn and spring, high summer temperatures and extreme events are triggering negative consequences. It remains to be seen how continued warming and increased atmospheric and soil dryness will affect forest resilience and the forest C sink, especially given the extreme events and negative consequences already observed in summer. We highlighted the complex interactions between phenology and CO_2_ fluxes across scales, showing that trees and forest respond similarly to abiotic drivers, albeit with different timing. These findings underscore the importance of considering seasonal dynamics and nonlinear ecosystem responses when assessing forest carbon budgets. Understanding the long‐term dynamics of forest responses to climate change is essential for predicting their future role in the global carbon cycle and for informing strategies to mitigate the impacts of climate change. While the Davos forest has contributed to climate protection in the past, its ability to maintain this role under future conditions remains uncertain.

## Author Contributions

**Luana Krebs:** conceptualization, formal analysis, investigation, methodology, visualization, writing – original draft. **Lukas Hörtnagl:** data curation, formal analysis, investigation, writing – review and editing. **Liliana Scapucci:** methodology, writing – review and editing. **Mana Gharun:** conceptualization, investigation, methodology, writing – review and editing. **Iris Feigenwinter:** investigation, visualization, writing – review and editing. **Nina Buchmann:** conceptualization, funding acquisition, investigation, methodology, project administration, supervision, validation, writing – review and editing.

## Conflicts of Interest

The authors declare no conflicts of interest.

## Supporting information


**Data S1:** gcb70371‐sup‐0001‐Supinfo.pdf.

## Data Availability

The data that support the findings of this study are openly available in ETH research collection at https://doi.org/10.3929/ethz‐b‐000597213.
