# Supplementary material for: Net Ecosystem CO2 Exchange of a Subalpine Spruce Forest in Switzerland Over 26 Years: Effects of Phenology and Contributions of Abiotic Drivers at Daily Time Scales
Source: Glob Chang Biol. 2025 Jul 24;31(7):e70371. doi: 10.1111/gcb.70371 (PMC12287681; doi:10.1111/gcb.70371)
Supplement: Supplementary file 1 — Data S1: gcb70371‐sup‐0001‐Supinfo.pdf. [file GCB-31-e70371-s001.pdf]

Appendix A. Additional figures and tables

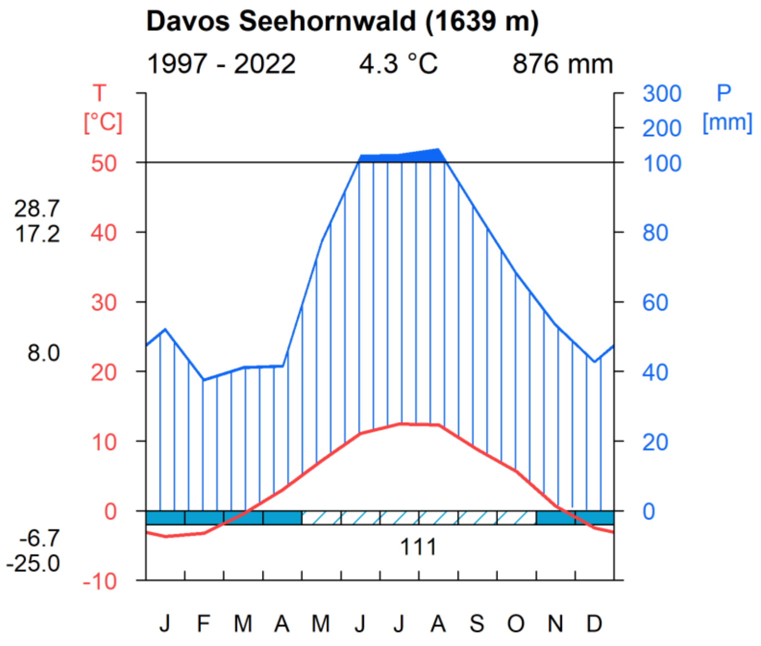

Fig. A1: ***Climate chart for the site CH-Dav for the years 1997 to 2022 (after*** *Walter and Lieth 1967)****. M****ean monthly air temperature (red line) and mean monthly precipitation sum (blue line) are given. The humid period of the year is shown as the blue vertically hatched area, the months with precipitation greater than 100 mm are indicated by the blue filled area (note: axis scale changes > 100 mm). Average annual temperature and average annual precipitation sum are listed at the top. Maximum measured temperature (from half-hourly values), mean daily maximum temperature of the warmest month (July), mean annual temperature amplitude, mean daily minimum temperature of the coldest month (January) and minimum measured temperature are given to the left. Petrol shaded areas on the x axis indicate months when mean daily minimum temperature is <0 °C; petrol hatched areas indicate months with absolute minimum temperature ≤0 °C (frost possible). The number in the middle of the x axis states the mean duration of consecutive frost-free days (when daily mean temperature does not drop below 0).*

Tab. A1: List of instruments used at CH-Dav over the 26 years of measurements during 1997 to 2022.

Variable	Type	Period
**Sonic anemometer**	Solent R2, Gill Instruments Ltd, Lymington (Hampshire) UK	01.01.1996 – 20.12.2006
	Solent R3-50, Gill Instruments Ltd, Lymington (Hampshire) UK	20.12.2006 – 17.11.2016
	HS-50, Gill Instruments Ltd, Lymington (Hampshire) UK (HS-50 Head_140505)	16.07.2014 – 22.09.2020<br>26.02.2021 – 08.03.2022
	HS-100, Gill Instruments Ltd, Lymington (Hampshire) UK (HS-100 Head_H000027)	22.09.2020 – 26.02.2021<br>08.03.2022 – 31.12.2022
**Infrared gas analyzer**	Closed path IRGA LICOR 6262, Licor, Lincoln (Nebraska) USA	01.01.1996 – 09.08.2005<br>Calibrations approx. 1-3 times per year
	Open path IRGA LICOR 7500, Licor, Lincoln (Nebraska) USA (serial Nr.75H-0639)	09.08.2005 – 07.10.2016<br>Calibrations approx. 1-2 times per year
	Enclosed path IRGA LICOR 7200, Licor, Lincoln (Nebraska) USA (LI-7200 Head serial Nr. 72H-0215)	25.09.2012 – 10.06.2020<br>Calibrations approx. 1-3 times per year
	Enclosed path IRGA LICOR 7200, Licor, Lincoln (Nebraska) USA (LI-7200 Head serial Nr. 72H-0586)	10.06.2020 – 05.11.2020
	Enclosed path IRGA LICOR 7200, Licor, Lincoln (Nebraska) USA (LI-7200 Head serial Nr. 72H0215)	05.11.2020 – 08.11.2021
	Enclosed path IRGA LICOR 7200RS, Licor, Lincoln (Nebraska) USA	08.11.2021 – 14.10.2022

	(LI-7200RS Head serial Nr. 72H-0834)	
	Enclosed path IRGA LICOR 7200RS, Licor, Lincoln (Nebraska) USA<br>(LI-7200RS Head serial Nr. 72H0585)	14.10.2022 – 31.12.2022
**Radiation budget**	CNR1, Kipp & Zonen B.V., Delft, The Netherlands	01.01.1996 – 01.11.2013
	CNR1, Kipp & Zonen B.V., Delft, The Netherlands<br>(CNR1_020518)	01.11.2013 – 23.05.2019
	CNR4, Kipp & Zonen B.V., Delft, The Netherlands<br>(CNR4_160819)	12.04.2017 – 14.10.2022
	CNR1, Kipp & Zonen B.V., Delft, The Netherlands<br>(CNR1_020522)	14.10.2022 – 31.12.2022
**Photosynthetic photon flux density**	PAR Quantum Sensor SKP 215, Skye Instruments Ltd, Powys, UK<br>(PAR LITE_050602)	10.07.2000 – 24.09.2015
	PAR LITE, Kipp & Zonen B.V., Delft, The Netherlands (PAR LITE_050602)	24.09.2015 – 31.12.2022
	PAR LITE, Kipp & Zonen B.V., Delft, The Netherlands (PAR LITE_050611)	24.09.2015 – 31.12.2022
	BF5 Sunshine Sensor, Delta-T Devices Ltd., London, UK<br>(BF5_8102)	31.01.2018 – 14.10.2022
**Air temperature and relative humidity**	HygroClip HC2, ROTRONIC AG, Bassersdorf, Switzerland<br>(HC2 S3_0060883608)	02.09.2014-10.12.2019
	HygroClip HC2, ROTRONIC AG, Bassersdorf, Switzerland<br>(HC2 S3_0020072927)	10.12.2019- 12.02.2020<br>17.03.2020 – 31.12.2022
**Precipitation**	1518 H3, LAMBRECHT meteo GmbH, Göttingen, Germany	01.01.1997 – 31.12.2022
**Soil water content**	EC-5, Decagon Devices, Inc., Pullman, WA, USA	01.01.2007 – 31.12.2022

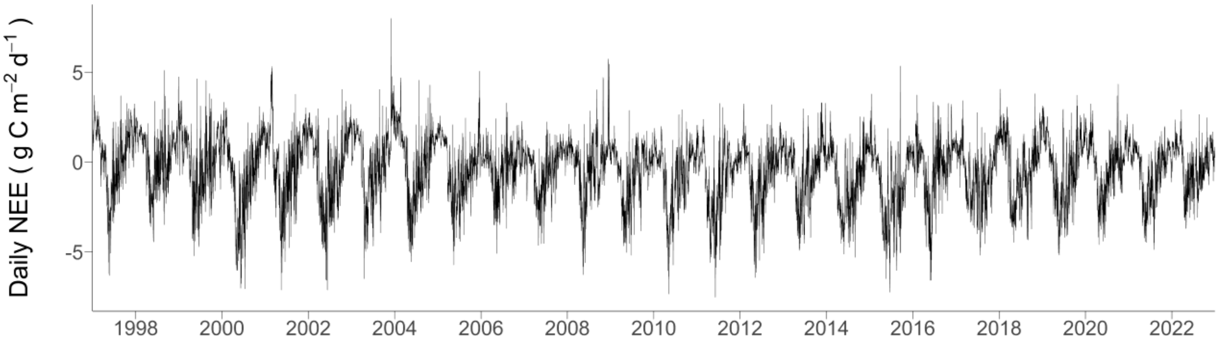

Fig. A2: Time series of daily net ecosystem CO_2 exchange (NEE) at CH-Dav during the 26 years of this study (1997-2022). NEE is given after quality checks and gap-filling (see text for details).

Tab. A2: Predictor variables (n=21) included in the final XGBoost model to predict mean daily NEE for the period 1997-2022.

	Original variable	Smoothed versions (moving average, MA)	Cumulative versions (moving sum, MS)	Lagged versions (moving variable forward in time)
Daily mean T_{air} (T_{air_mean})	x	3-day MA ($T_{air\_mean\_MA3}$)	-	$T_{air\_mean\_MA30}$ with 180-day lag ($T_{air\_mean\_MA30\_lag180}$)<br>$T_{air\_mean\_MA30}$ with 365-day lag ($T_{air\_mean\_MA30\_lag365}$)
Daily minimum T_{air} (T_{air_min})	x	5-day MA ($T_{air\_min\_MA5}$)<br>30-day MA ($T_{air\_min\_MA30}$)	-	-
Daily maximum T_{air} (T_{air_max})	x	-	-	-
Number of hours with T_{air} <0 °C ($T_{air<0}$)	-	-	15-day MS ($T_{air<0\_MS15}$)<br>90-day MS ($T_{air<0\_MS90}$)	$T_{air<0\_MS15}$ with 15-day lag ($T_{air<0\_MS15\_lag15}$)
Short-wave radiation (Rg)	x	30-day MA (Rg_{MA30})	-	Rg_{MA30} with 180-day lag (Rg_{MA30_lag180})
Photosynthetic photon flux density (PPFD)	x	-	-	-

Daylength	x	-	-	-
Precipitation (Prec)	x	-	30-day MS ($Prec_{MS30}$)	-
Soil water content (SWC)	-	15-day MA (SWC_{MA15})	-	-
Relative humidity (RH)	x	-	-	-
Vapour pressure deficit (VPD)	x	-	-	-

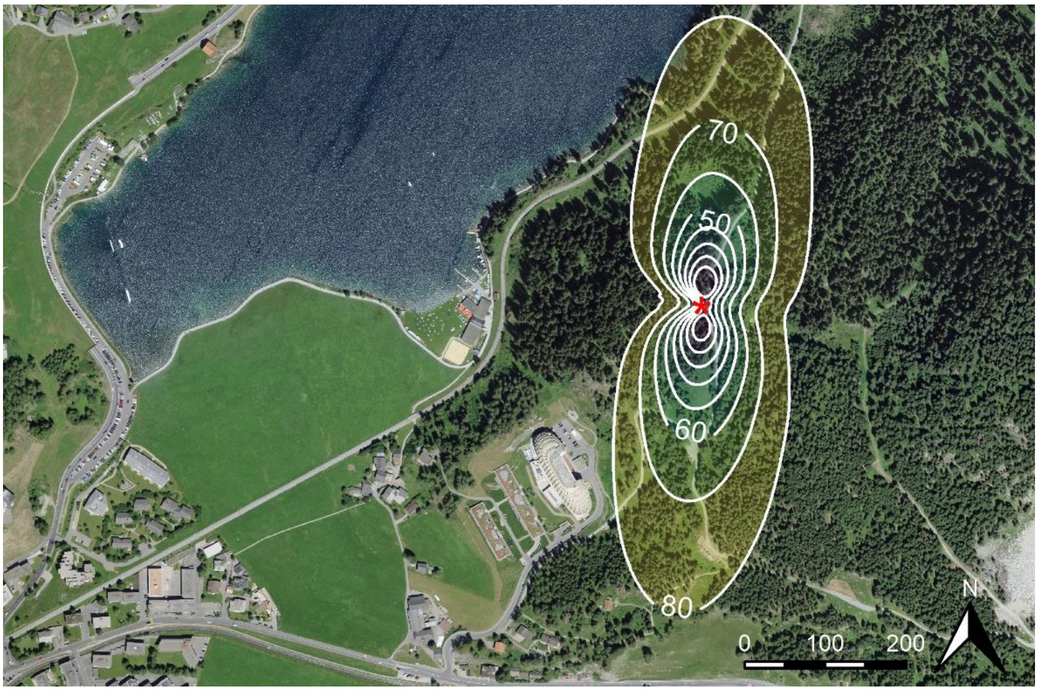

Fig. A3: Footprint area of the flux station at CH-Dav. Footprint was estimated for the year 2022 with the Kljun et al. (2015) footprint model. Contour lines represent the area within the footprint where 80, 70, 60, 50, 40, 30, 20, and 10% of the signal measured at the station (red asterisk) originate. The background orthophoto is from the Federal Office of Topography swisstopo.

Tab. A2: Details on hyperparameters used in the XGBoost model. Hyperparameters and effects on the model are described in Chen et al. (2024).

Hyperparameter	
nrounds	2500
early_stopping_rounds	50
eta	0.03
max_depth	4
gamma	14
min_child_weight	3
colsample_bytree	1
subsample	0.5

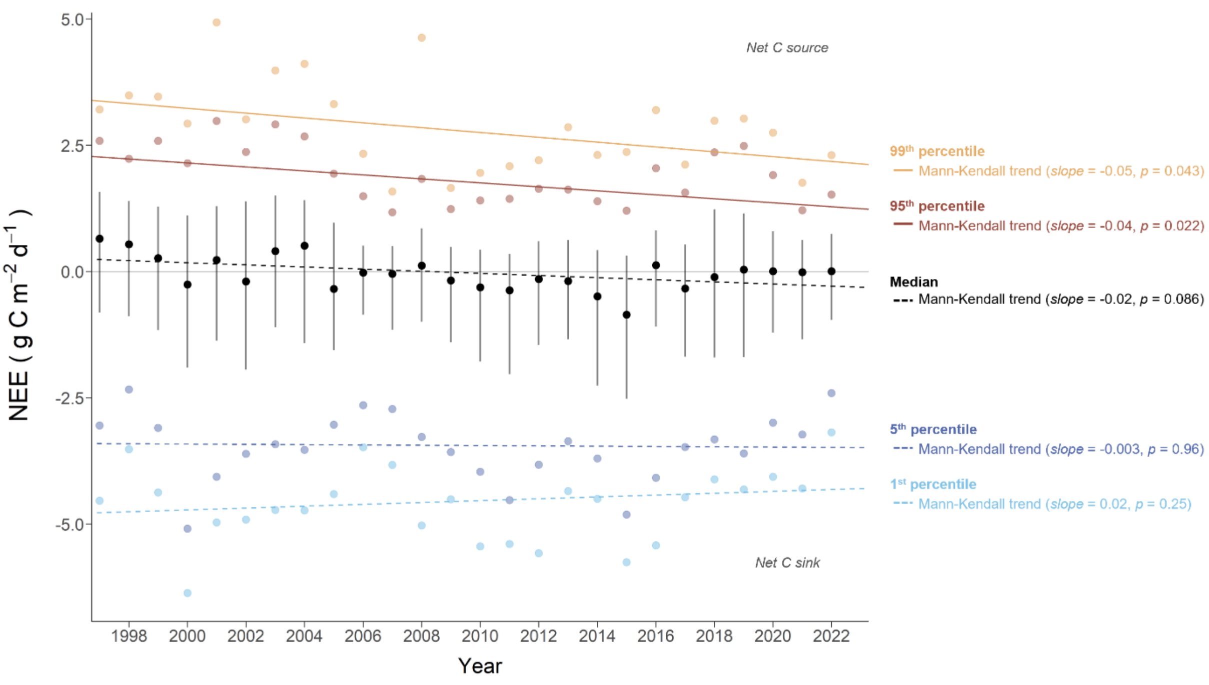

Fig. A4: Daily cumulative $NEE_{c,DOY}$ of the spruce forest CH-Dav, per year, during 1997 to 2022. Median $NEE_{c,DOY}$ per year (black dots with error bars, 1st and 3rd quartile of measured $NEE_{c,DOY}$), peak C uptake days per year (light and dark blue circles, 1st and 5th percentile $NEE_{c,DOY}$ per year), and peak C loss days per year (orange and red circles, i.e., 95th and 99th percentile daily $NEE_{c,DOY}$ per year) are shown. The solid and dashed lines depict the Mann-Kendall trends (solid lines: $p<0.05$; dashed lines: $p>0.05$).

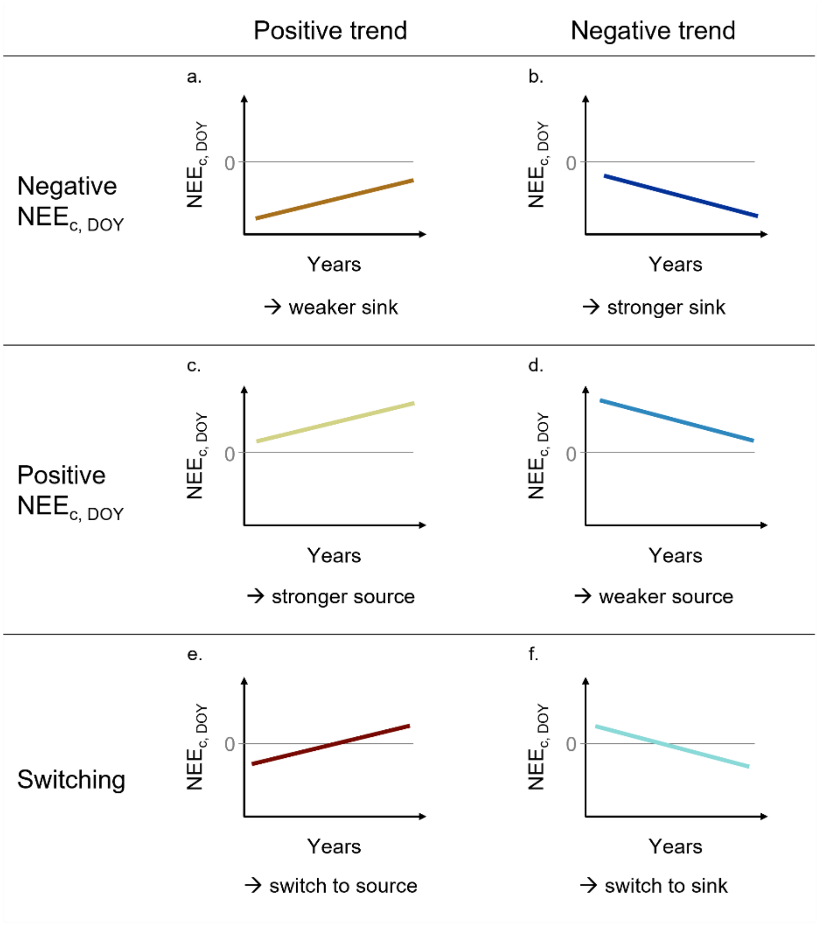

Fig. A5: Conceptual figure showing the six potential trends in $NEE_{c,DOY}$. a) positive trend in negative $NEE_{c,DOY}$ indicating a trend to a weaker C sink, b) negative trend in negative $NEE_{c,DOY}$ indicating a trend to a stronger C sink, c) positive trend in positive $NEE_{c,DOY}$ indicating a trend to a stronger C source, d) negative trend in positive $NEE_{c,DOY}$ indicating a trend to a weaker C source, e) trend showing a switch from negative to positive $NEE_{c,DOY}$ indicating a switch from C sink to C source, f) trend showing a switch from positive to negative $NEE_{c,DOY}$ indicating a switch from C source to C sink.

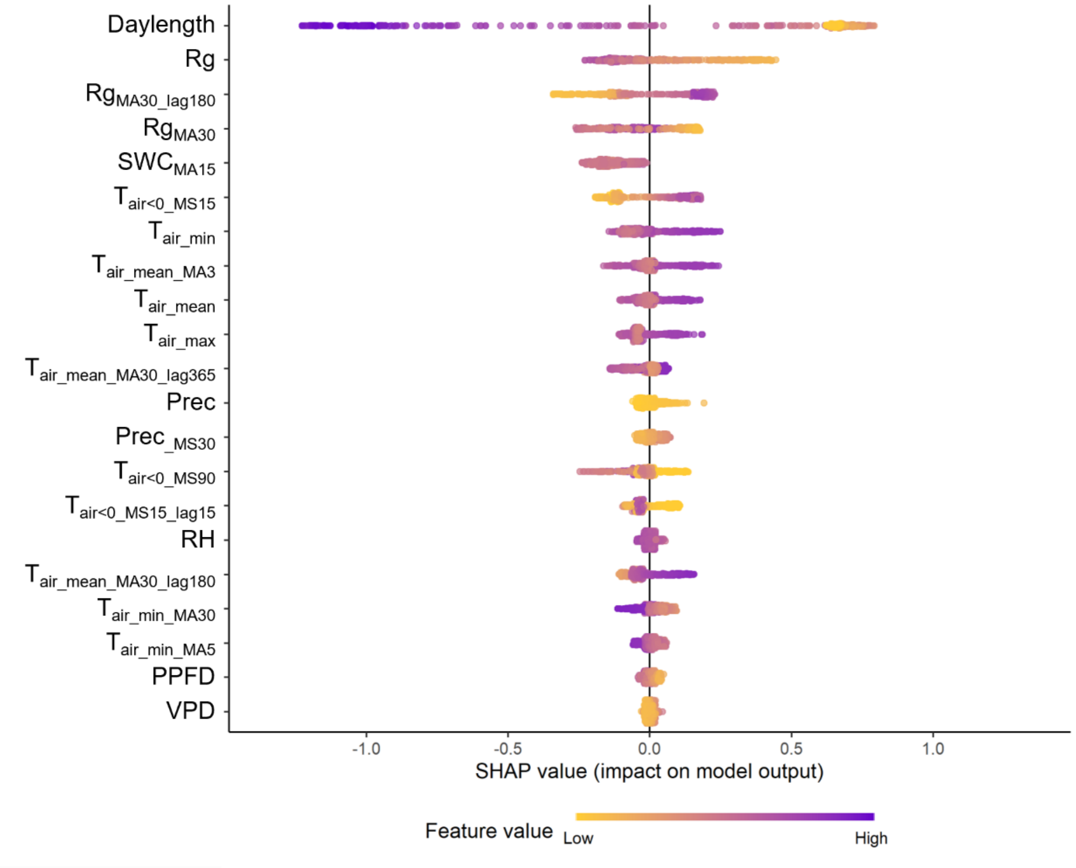

Fig. A6: Beeswarm plot showing SHAP values of all model features for the prediction of daily NEE of CH-Dav over 26 years. Features are ordered from the most to the least important driver variable, colored according to feature values (yellow for low and purple for high values). For abbreviations, see Tab. A2.

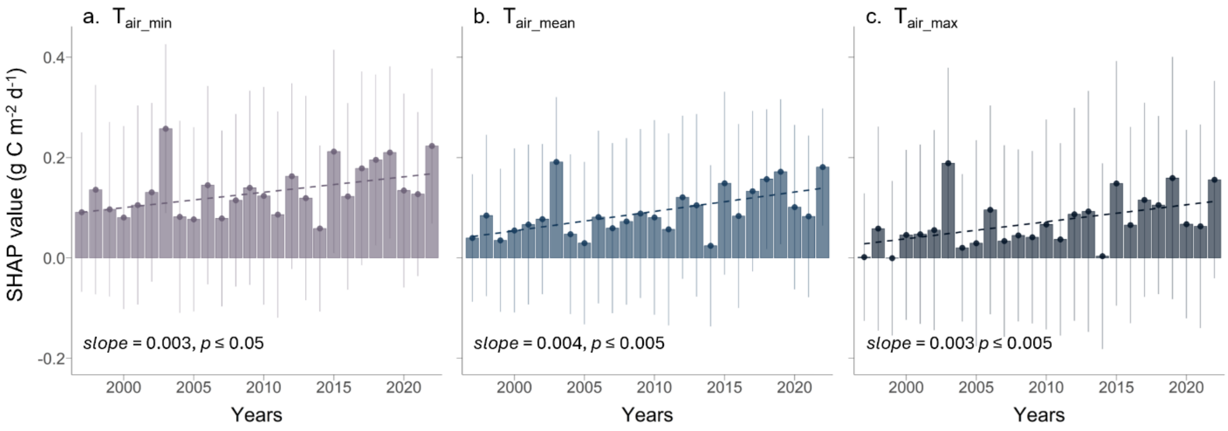

Fig. A7: Mean SHAP values for a) T_{air_min}, b) T_{air_mean}, c) T_{air_max} in the summer months (JJA) to predict daily cumulative NEE ($NEE_{c,DOY}$) between 1997 and 2022. Mean and standard deviation (SD) of the SHAP values are given as error bars. Note the large effect of all temperatures in the year 2003, with very hot and dry conditions in Europe (Domeisen et al., 2023).
